# Embracing the Versatility of Botulinum Neurotoxins in Conventional and New Therapeutic Applications

**DOI:** 10.3390/toxins16060261

**Published:** 2024-06-04

**Authors:** Christine Rasetti-Escargueil, Stefano Palea

**Affiliations:** 1 The Institute of Cancer Research, 237 Fulham Road, London SW3 6JB, UK; 2 Humana Biosciences-Prologue Biotech, 516 Rue Pierre et Marie Curie, 31670 Labège, France; stefano.palea@humana-biosciences.com

**Keywords:** botulinum neurotoxins, toxinotypes, subtypes, engineering, therapy

## Abstract

Botulinum neurotoxins (BoNTs) have been used for almost half a century in the treatment of excessive muscle contractility. BoNTs are routinely used to treat movement disorders such as cervical dystonia, spastic conditions, blepharospasm, and hyperhidrosis, as well as for cosmetic purposes. In addition to the conventional indications, the use of BoNTs to reduce pain has gained increased recognition, giving rise to an increasing number of indications in disorders associated with chronic pain. Furthermore, BoNT-derived formulations are benefiting a much wider range of patients suffering from overactive bladder, erectile dysfunction, arthropathy, neuropathic pain, and cancer. BoNTs are categorised into seven toxinotypes, two of which are in clinical use, and each toxinotype is divided into multiple subtypes. With the development of bioinformatic tools, new BoNT-like toxins have been identified in non-Clostridial organisms. In addition to the expanding indications of existing formulations, the rich variety of toxinotypes or subtypes in the wild-type BoNTs associated with new BoNT-like toxins expand the BoNT superfamily, forming the basis on which to develop new BoNT-based therapeutics as well as research tools. An overview of the diversity of the BoNT family along with their conventional therapeutic uses is presented in this review followed by the engineering and formulation opportunities opening avenues in therapy.

## 1. Introduction

The first clinical applications of BoNTs were envisioned by Dr Justinus Kerner in 1815–1817 when he proposed the use of this “sausage toxin” to treat hypersecretion disorders. Dr Justinus Kerner was a German physician (as well as Romantic poet and polymath) who established the link between flaccid paralysis and the consumption of spoiled sausages. Dr Kerner had investigated numerous “sausage poisoning” cases following blood sausages consumption in the southwest region of Germany. He then established that an unknown toxin, able to develop under anaerobic conditions in the blood sausages was lethal in minute doses by causing descending flaccid paralysis. Dr Kerner formulated for the first time the therapeutic potential of this sausage poison thanks to its ability to inhibit muscle tonicity and alleviate hypersecretion or neurological disorders resulting from muscle overactivity. In 1895, Van Ermengen identified the causative agent, *Bacillus botulinum*, at this stage renamed the “*Clostridium botulinum*”, isolated during a severe outbreak that killed three musicians in a Fanfare in Belgium, while many others became seriously ill. To complete his findings, Van Ermengen found that the culture filtrates administered to animals induced botulism and led to death [1,2,3]. Nevertheless, it took almost a century before Dr Alan Scott discovered the benefit of botulinum toxin (Oculinum©) to treat strabismus [4]. Nowadays, a wide range of potential therapies remain unexploited in complement to the present formulations exclusively based on the first two identified toxinotypes, namely BoNT/A and BoNT/B. Furthermore, genetically engineered botulinum toxin products are now in development to further expand therapeutic uses [5].

BoNTs form a family of neurotoxins produced by spore-forming Gram-positive anaerobic bacteria named *Clostridium botulinum* and *Clostridium* spp., such as *Clostridium butyricum*, *Clostridium argentinensis*, and *Clostridium baratii* [6,7]. Botulinum neurotoxins are part of the “dirty dozen” agents listed as bioweapons by the CDC [8]. All *Clostridium botulinum* strains were historically classified into four distinct metabolic groups (I–IV) according to their biochemical properties and their physiology: saccharolytic and proteolytic abilities, alcohol fermentation products, heat resistance of their spores, and ability to grow in acids, salts, and alcohol at different temperatures [3,9]. Rare but often severe intoxication by BoNTs, or toxi-infection by these bacteria, results in a clinical condition called botulism, or intestinal botulism, due to intestinal colonisation by *Clostridium* spores, respectively. Flaccid paralysis may be fatal if the patient has no access to intensive care. Unfortunately, the treatment of botulism nowadays remains mainly symptomatic, including assisted ventilation within the intensive care unit for severe cases.

BoNTs are 150 kDa modular proteins consisting of two peptide chains connected by a disulfide bond: a 100 kDa heavy chain (HC) and a 50 kDa light chain (LC) [10]. The translocation domain consists of a 50 kDa chain at the N-terminal domain (HN). This domain is involved in the translocation of the LC, containing a metalloprotease, into the intracellular compartment, after the receptor binding domain of the BoNT has bound to an outer membrane receptor of the target neuronal cell [11,12]. The peptidic chains of BoNTs present significant differences along their amino acid sequences among each subtypes [13]. The C-terminal domain (HC), is composed of two sub-domains (HCn and HCc). This HC domain is involved in the binding of the toxin to neuronal membranes at specific presynaptic receptor sites. The delivery of the LC into neuronal cells through the vesicle membranes is subsequently mediated by the translocation domain situated on the N-terminal domain of the heavy chain (HN) to facilitate its entry into the neuronal cytosol. The HN is a helical bundle that protects and escorts the LC across endosomal membranes. This protein-conducting channel is actioned by the transmembrane proton gradient that supports LC cargo unfolding during translocation and finally triggers the release and refolding of the LC in the cytosol [14,15]. The modular architecture of BoNT is such that each BoNT module functions individually while each domain serves as a chaperone for the others. The receptor binding domain facilitates the unfolding of the LC to reach a translocation permissive conformation in synchrony with the formation of the translocation channel formation. The modular function of the translocation domain makes BoNT an attractive tool for molecules delivery in target tissues. By replacing the neuronal targeting binding domain with one domain recognising a specific surface protein could transform the BoNTs into delivery system targeting specific tissues [16]. The HCn subdomain is involved in toxin binding by interacting with lipid microdomains [17,18,19].

The BoNT holotoxins are produced as non-covalently bound complexes of different protein components forming the progenitor toxin complexes (PTCs) [20]. The progenitor toxin complex is composed of non-toxic neurotoxin-associated proteins (NAPs) such as hemagglutinins (HA-17, HA-33, and HA-70) and a non-toxic non-hemagglutinin (NTNHA) protein. These auxiliary proteins (HAs and NTNHAs) ensure appropriate stabilisation, preservation, and absorption of the BoNTs during the intoxication process [21,22,23].

BoNTs are initially produced as single chain proteins undergoing post-translational proteolytic activation via environmental or host proteases into a disulfide-linked ~50 kDa light chain (LC) and ~100 kDa heavy chain (HC) di-chain protein before reaching the host [24,25]. BoNT/E is produced as a single chain toxin and later cleaved by the host proteases into the di-chain form which is about 100-fold more toxic than the single chain form. This cleavage step shows that di-chain formation is an essential step of the BoNT activation process [26].

The BoNTs enter synaptic terminals and cleave one of the three soluble N-ethylmaleimide-sensitive factor attachment protein receptor (SNARE) proteins, namely vesicle-associated membrane protein (VAMP), synaptosomal-associated protein 25 (SNAP25), and syntaxin, leading to the inhibition of neurotransmitter release by synaptic vesicles [14]. Botulism can last for months and require extensive medical treatment. The only available therapy is antitoxin administration, only effective if administered early before toxin entry into the neuronal cells [27]. Besides this shared mechanism of neurotransmitter release disruption, the intracellular targets and receptors, the pharmacodynamic/kinetic properties greatly vary between the various BoNT toxinotypes.

BoNTs are categorised into at least seven toxinotypes, termed A–G, two of which are in clinical use, types A and B. Each toxinotype is divided into multiple subtypes using a number defined by amino acid sequence differences >2.6% [28]. In 2014, an eighth novel BoNT, called BoNT/H, was identified by Arnon and colleagues in clinical isolates, on the basis of sequence analysis and its lack of neutralisation by sera against known toxinotypes [29]. Nevertheless, this toxinotype was later identified as BoNT/FA or HA mosaic toxin, named BoNT/H or BoNT/FA, since its mosaic structure comprised regions of similarity with BoNT/A and F and BoNT/A antitoxin was able to neutralise it. Its LC showed similarities to BoNT/F5 and the HC presented similarities with BoNT/A1-HC [30,31]. 

More recently, with the development of bioinformatic tools, new BoNT-like toxins have been reported and characterised, including BoNT/X in a *Clostridium botulinum* strain [32,33]. New BoNT/like sequences were identified in non-clostridial species such as *Weissella oryzae* and *Chryseobacterium piperi*, BoNT/En protein was found in an *Enterococcus faecium* strain [33], Paraclostridial Mosquitocidal Protein 1 (PMP1) was found in the *Paraclostridium bifermentans* strains [34], and PGT1/2 protein was found in *Paeniclostridium ghonii* [35]. Among those BoNT-like proteins, BoNT/X cleaves VAMP1-3 at a unique site, and it cleaves VAMP 4 and 5, as well as Ykt6 (a VAMP family protein). LC/X is also ~10-fold more efficient at the cleavage of VAMP1 [26,36,37]. However, the native BoNT/X produced by *C. botulinum* Strain 111 and the recombinant form of BoNT/X exhibit very weak potency in human-induced pluripotent stem cell-derived neuronal cells and in the mouse model [38]. The other non-clostridial LC/En cleaved VAMP 1-3, Syntaxin (Syx 1B and Syx 4), as well as SNAP-23 and SNAP-25 but at lower efficiency for SNAP-25, Syx 1B, and Syx 4 [26]. The LC/Wo cleaves VAMP-2 [39]. Understanding the complexity of BoNTs and BoNT-producing clostridia nomenclature represents one crucial challenge to ensure accurate reporting in diagnostic settings, in food safety testing and evaluation of therapeutic BoNTs. For example, it is paramount to distinguish between Botulinum strains associated with a human botulism case, an animal outbreak, a food safety risk, or BoNT therapeutic formulation analysis [40].

Because of the known variability of BoNT-derived products, a summary of the therapeutic profiles of the conventional BoNT formulations will be presented in this review, in parallel to more recent formulations involving engineered BoNTs and exploiting the versatility of the BoNTs to develop new injection-free or local formulations.

## 2. Overview of Conventional Therapeutic Applications and Formulations

A few years after Dr Alan Scott’s discovery showing that BoNT/A could relax eye muscles and treat strabismus, the first approved BoNT/A formulation was licensed in 1984 for human therapeutic use.

In the 1970s, corrective surgery to treat strabismus required improvements since most patients needed reoperation. Dr Scott, an ophthalmologist, was testing substances able to weaken eye muscles and realign them. To this aim, he experimentally injected Botulinum toxin A into eye muscle on monkeys. He found that muscle weakness induced by BoNT/A was specific and prolonged, causing no local side effects nor systemic toxicity [41]. He then formulated the Botulinum toxin A produced by Dr. Edward Schantz, who was a microbiologist in the Johnson laboratory at the University of Wisconsin, to become a safe formulation for injection into human patients. The toxin stabilising agent was changed from the gelatin to human serum albumin since it was approved for human use. Dr Scott’s team soon established that the toxin amounts were to be expressed based on the mouse LD50 test to assess its actual biological activity rather than measure a mass of protein. After successfully treating strabismus in humans, Dr Scott and his collaborators used BoNT/A to treat blepharospasm and torticollis patients. Subsequently, Dr Joseph Tsui, a neurologist, injected the toxin to treat multiple sclerosis patients presenting with spasticity. The product from the Schantz and Johnson laboratory was registered and manufactured until the late 1970s through the Oculinum company under the name Oculinum^®^ [42]. Oculinum^®^ was subsequently distributed by Allergan, Inc. to meet the growing clinical demand [43]. The beneficial effects of BoNT/A on facial glabellar lines were observed by chance when ophthalmologists treated patients suffering from involuntary blinking. The Allergan company modified the brand name of Oculinum^®^ to Botox^®^ after the finalisation of the acquisition of Oculinum, Inc. company in 1991. 

Meanwhile, the Public Health Laboratory Service in the United Kingdom developed a different BoNT/A product. In 1992, this BoNT/A product was licensed for Europe with the brand name Dysport^®^ and non-proprietary name “abobotulinumtoxinA”. Then, Ipsen France acquired the company and subsequently sold the cosmetic operations to Galderma (Lausanne, Switzerland) who renamed the Dysport^®^ product as Azzalure^®^ [44,45]. Given that only minute doses of BoNTs are sufficient to silence neuromuscular junctions as well as modulate sensory fibres, natural BoNTs represent an ideal therapeutic repertoire. Moreover, only a small proportion of patients may develop resistance due to the production of antibodies following repeated injections of BoNT. In addition to its extreme potency, the effects of BoNT/A can last for more than 3–6 months in humans.

### 2.1. A Wide Range of Conventional Therapeutic Applications

Dr. Jankovic, who was also a pioneer in using BoNT injections into patients to treat blepharospasm, led and published the first double-blind, placebo-controlled trial focused on the treatment of cranial–cervical dystonia by BoNT [46]. Those trials were instrumental to obtain approval by the Food and Drug Administration (FDA) in 1989 for BoNT formulations to treat blepharospasm, hemifacial spasm (HFS), and other types of facial nerve disorders [47]. BoNTs have been given regulatory approval for the treatment of many disorders caused by muscle over-contractility. Therefore, BoNTs are routinely used to treat movement disorders like dystonia or spasticity. Regarding movement disorders originating from neurologic diseases, BoNT was proven to be effective in patients with bruxism, tremors, tics, myoclonus, restless legs syndrome, and tardive dyskinesia. BoNT has been shown to be effective against common disorders observed in Parkinson’s disease such as foot dystonia, freezing of gait, rigidity, and tremor [45].

Recent studies have expanded the use of BoNT from a powerful muscle relaxant in the periphery to the relief of neurodegenerative disease originating from the central nervous system. In addition to muscle hyperactivity, BoNTs are also efficient to treat hypersecretion disorders like hyperhidrosis and sialorrhea [48,49].

Furthermore, BoNTs represent a unique therapeutic opportunity to modulate neuronal function since they are reported to affect inhibitory, excitatory, and sensory neurons [50]. Experimental observations show that BoNT is involved in the modulation of sensory feedback loop going to the central nervous system and resulting in analgesia. The therapeutic benefit of BoNT has been shown in chronic migraine [51]. As a result, the use of BoNTs to reduce pain has increased significantly, suggesting an expanding range of applications in chronic pain. BoNTs have been shown to inhibit the release of glutamate, substance P (SP), and calcitonin gene-related peptide (CGRP) acting as pain neurotransmitters by altering synaptic vesicle fusion and transient receptor potential (TRP) channels at the neuronal membrane [52]. Dr Aoki and coworkers established that nociception inhibition by BoNT/A was due to the alteration of the mechanosensitive ion channels fusion into the nerve terminals of peripheral trigemino-vascular neurons. In the treatment of migraine headache, the action of BoNT/A is due to the blockade of neurotransmitters release as well as inhibition of inflammatory peptides release and relevant cell surface ion channels expression [53].

Besides their targeting of neurons, the effects of BoNTs were evidenced in human skin restoration and other tissues, which will expand the therapeutic uses of BoNTs [54]. Studies showing that BoNT/A can induce skin cell restoration and improve global skin condition augur a wider impact of BoNTs than exclusive SNARE cleavage at neuronal vesicles. A range of experimental studies show the protective effects of BoNTs on skin flaps, wound healing, hypertrophic scars, and psoriasiform dermatitis, and wider effects like anti-inflammatory and anti-cancer effects [54]. Injections of BoNT-A have been shown to reduce signs and symptoms of acne, rosacea, and psoriasis and to bring about significant improvements in several rare diseases that are caused or exacerbated by hyperhidrosis [55]. More recently, experimental studies have evidenced the beneficial effects of BoNT/A in alopecia after artificially inducing hair loss in the mouse under continuous stress conditions [56].

### 2.2. Variability in the BoNT Formulations

The available therapeutic formulations of BoNT currently approved for use in human in the USA and in Europe contain only the two main toxinotypes of BoNT, namely botulinum toxin type A (BoNT/A) or botulinum toxin type B (BoNT/B): abobotulinumtoxinA (ABO, Dysport^®^ product from Ipsen, Les Ulis, France), incobotulinumtoxinA (INCO, Xeomin^®^ or Bocouture^®^ products from Merz Pharmaceuticals GmbH, Frankfurt, Germany), onabotulinumtoxinA (ONA, Botox^®^ product from Abvie/Allergan, Irvine, CA, USA), and rimabotulinumtoxinB (Myobloc^®^ from Solstice Neurosciences now Supernus Pharmaceuticals, Rockville, MD, USA) (see Table 1) [57]. Currently, BoNTs are employed for an expanding number of indications associated with muscle overactivity and for cosmetic purposes, in more than 20 therapeutic formulations for almost 20 therapeutic indications (see Table 1 for timelines of approval of each formulation). The following ratios were calculated, based on initial studies, in order to achieve a similar efficacy in clinical practice: onabotulinumtoxinA versus incobotulinumtoxinA = 1; onabotulinumtoxinA versus abobotulinumtoxinA = 1:2.5; onabotulinumtoxinA versus rimabotulinumtoxinB = 1:50; these ratios show that the different BoNT formulations are not interchangeable [58]. A significant number of clinical studies have confirmed that ONA and INCO show therapeutic equivalence in a wide range of indications notably cervical dystonia and blepharospasm, whereas between ONA and ABO formulations, the application of a predetermined conversion ratio (close to 1:25) was recognised as being the most appropriate [59].

New BoNT formulations are under development, but with only a few being FDA-approved thus far. DaxibotulinumtoxinA (Daxxify^®^) is a new formulation of BoNT/A for cervical dystonia which showed a median effect duration of 24 weeks at doses of 125 U. The DaxiBoNT-A formulation contains a stabilising peptide that ensures longer-lasting effects and a shelf-life of two years. The DaxiBoNT-A formulation does not contain animal-derived components nor human albumin and does not require refrigeration. The evaluation of the safety, duration of response, and efficacy of two doses of DaxibotulinumtoxinA for the treatment of cervical dystonia (CD) served as the foundation for the Food and Drug Administration (FDA) approval of Daxxify^®^ in August 2023 [60,61]. This prolonged duration of action represents a significant step forward from previous formulations since this will allow a longer inter-visit interval than the usual interval of about 3 to 4 months. However, it should be mentioned that the prolonged effect may also be due to the higher doses of Daxxify^®^ injected. The LanbotulinumtoxinA (Lantox ^®^ product, Lanzhou, China) is a more classical formulation of BoNTA manufactured and registered in China since 1994. Despite its widespread use in China and other Asian countries and in South America, it is not yet approved elsewhere [62].

**Table 1:** History of botulinum neurotoxin (BoNT) research and clinical development.

1822	Justinus Kerner	Sausage poison (envisioned therapeutic potential)
1870	Müller	Disease called “botulism” (Latin: *botulus* meaning sausage)
1895	Van Ermengem	*Clostridium botulinum* (microorganism causing botulism)
1919	G.S. Burke	Minimum lethal dose established in guinea pigs
1928	Herman Sommer	Isolation and purification of BoNT
1946	Carl Lamanna Edward Schantz	Neurotoxin activity determined using the LD50 test BTA produced in the crystalline form
1949	Arnold Burgen	BoNT induces the blockade of the neuromuscular transmission
1950	Vernon Brooks	BoNT/A: -blockade of acetylcholine from motor nerve endings
1960s	Schantz/Scott	BoNT/A to treat strabismus in monkeys
1980	Scott	BoNT/A to treat strabismus in humans
1987	Drs. Jean and Alastair Carruthers	Ophthalmologists treating patients for involuntary blinking discover the cosmetic benefits of BoNT/A
1988	Allergan	First clinical trial on Oculinum (BoNT/A)
1989	FDA Allergan	First official indications: strabismus, blepharospasm, hemifacial spasm, and dystonia Allergan buys and renames Oculinum as Botox^®^
1990	MHRA	Dysport^®^ (abobotulinumtoxinA, Ipsen) (~) approval for dystonia
1993	Montecucco and Schiavo	SNAP-25 is the molecular target of BoNT/A
1995	MHRA	Dysport^®^ (abobotulinumtoxinA, Ipsen, Wrexham, UK) approval for strabismus in the UK [63]
1997	China	Approval of Lantox^®^ (lanbotulinum toxin A, Lanzhou Institute of Biological Products, Lanzhou, China) for strabismus, blepharospasm, and hemifacial spasm.
1999	FDA	(New) Botox^®^ (onabotulinumtoxinA, Allergan, Irvine, CA, USA) approval for cervical dystonia
2000	FDA	First BoNT/B: NeuroBloc^®^ approved for cervical dystonia
2002	FDA	Botox^®^ approved for cosmetic uses as Botox Cosmetics^®^ (Australia, Switzerland, Taiwan, and Singapore) Lantox^®^ approval in Republic of Korea
2003	AFSSAPS	Botox product approved under Vistabel^®^ name (France)
2004	FDA	Botox^®^ approved for primary axillary hyperhidrosis
2005	EMA	Xeomin^®^ (incobotulinumtoxinA, Merz, Frankfurt, Germany) approved for blepharospasm and cervical dystonia in adults (Germany).
2006	MHRA	Botox^®^ approved under the name Vistabel^®^ for glabellar lines
2006	Korean FDA	Neuronox^®^ (MedyTox, Seoul, Republic of Korea) approved for blepharospasm (Meditoxin^®^, Botulift^®^, Cunox^®^)
2007	India	Approves Boto genie^®^ (Bio-med Ltd., Ghaziabad, India)
2009	MHRA FDA	Azzalure^®^ approval for treatment of glabellar lines Dysport^®^ (Abobotulinum) approved for glabellar lines and cervical dystonia
2010	FDA	Botox^®^ approval for adult upper limb spasticity Xeomin^®^ (Incobotulinum) approval for cervical dystonia and blepharospasm
2011	FDA	Botox^®^ approval for chronic migraine and urinary incontinence Xeomin^®^ (incobotulinumtoxinA) approval as Bocouture^®^ for glabellar lines in adult patients
2012	NHS UK China	Botox^®^ approval for chronic migraine Lantox^®^ approval for glabellar frown lines BoNT/A-specific cell-based potency assay to replace the mouse bioassay [64]
2013	Korean FDA FDA Japan	Nabota^®^ (Daewoongs Pharmaceuticals, Hwaseong, Republic of Korea) approval Evosyal^®^, Daewong/Evolus-Alphaeon R. Korea/USA Botox^®^ approval for overactive bladder Nerbloc^®^ (Eisai, Tokyo, Japan) approval
2014	China Korea	CBTX-A approved as Lantox^®^ and Prosigne^®^ (Lanzhou Institute of Biological Products, Lanzhou, China) (Hengli^®^, Lanzox^®^, CBTX-A^®^, Redux^®^, Liftox^®^, Dituroxal^®^) Botulax^®^, Zentox^®^, Regenox^®^ (Hugel Pharma, Gangwon-do, Republic of Korea)
2015	FDA EMA	Xeomin^®^ (incobotulinumtoxinA) and Dysport^®^ (AbobotulinumtoxinA) approval for adult upper limb spasticity Approves Bocouture^®^ for combined upper facial lines
2016	Korean FDA FDA	Approval of Coretox^®^ (Medytox, no complexing proteins, no biological excipients) Approval of Dysport^®^ for children lower limb spasticity
2017	FDA and EMA, Russia	Botox^®^ and Dysport^®^ approval for adult lower limb spasticity Relatox^®^ approval (Microgen, Moscow, Russia)
2018	FDA	Approval of Botox^®^ for forehead wrinkles and Xeomin^®^ for sialorrhea
2019	FDA	Approval of Botox^®^ for paediatric upper limb spasticity Approval of Jeuveau^®^ (Nabota) for glabellar lines Approval of Neuronox ^®^ for glabellar lines Approval of Botulax^®^ by PDUFA (Hugel R., Gangwon-d, Korea BT-A, Botox^®^ analogue)
2022	FDA	Approval of RT002 (DaxibotulinumtoxinA) for glabellar lines Revance Therapeutics (Nashville, TN, USA)
2022	Korean FDA	Masport^®^ Masoundarou I.R. Iran BT-A, Dysport^®^ analogue CosmeTox^®^ Transdermal USA BT-A, creme Lantox^®^ registered in Republic of Korea. EB-001 BONTi/Allergan (Irvine, CA, USA) BT-E
2023	FDA	MCL005 Malvern Cosmeceuticals UK BT-A, topic gel ANT-1207 Anterios/Allergan USA BT-A, lotion DaxibotulinumtoxinA (Daxxify^®^, Revance, Nashville, TN, USA) is a novel BoNTA preparation for cervical dystonia
2024	FDA	Approves Letybo^®^ (Hugel, Gangwon-do, Republic of Korea)

FDA: Food and Drug administration; MHRA: Medicines and Healthcare products Regulatory Agency; AFSSAPS: French Agency for the Safety of Health products. Modified from [62,63,64,65,66,67].

All BoNT-A formulations contain the 150 kD neurotoxin with neurotoxin-associated proteins (NAPs) except for INCO, which contains exclusively pure 150 kD neurotoxin. Nevertheless, the production process itself may impact the active toxin quantities, such as the addition of enzymes to increase the proportion of cleaved active toxin, which may cause the denaturation of the neurotoxin [68,69]. As a result, the injection units are variable among different formulations. Currently, ONA (Botox^®^) is available in 50/100/200 U vials, and ABO (Dysport^®^) is available in 300/500 U vials. Historically, it was estimated that the initial formulation of Botox^®^ contained a dose equivalent to 25–40 ng of BNT/A for 100 mouse LD50, while the formulation of Dysport^®^ contained 5 ng of BoNT/A for 500 mouse LD50. Regarding BoNT potency estimates, the variations in local BoNTs production processes as well as variability between in-house mouse bioassays needs to be considered. Therefore, there are differences in the pharmacodynamic and pharmacokinetic properties of the different formulations, which cannot be used equally [70] (see Table 2).

Each BoNT/A batch produced is specific to each manufacturer, and the final formulation varies between each product. While the mechanism of action of these products is the same, there are significant formulation disparities between them. The toxin potency in units is specific to each product, and not exactly equivalent since each company assesses their toxin batches using its own proprietary mouse LD50 assay (or cell-based assay). As a consequence, the “Dysport^®^ unit” is not exactly equivalent to the “Botox^®^ unit”, or a “Xeomin^®^ unit”. Moreover, the amount of human serum albumin also differs between the three formulations (see Table 2), which can influence the efficacy of each formulation in humans [71,72]. Several characteristics must be considered when administering botulinum neurotoxins since differences occur between formulations due to diverse manufacturing processes, bacterial strains used in fermentation, purification methods, and inactive ingredients in the formulation which influence the potency and antigenicity of the products. A range of clinical studies showed that the therapeutic and safety margins for Botox^®^ were larger than for Dysport^®^, due to a reduced tendency for Botox^®^ to cause undesired distant effects through diffusion related to its lower dosage. The adjustment in injection volumes, techniques, and patterns are required to achieve similar clinical results [72]. In addition to the variability between dose units, new formulations such as Prabotulinumtoxina A-xvfs, Neu-botulinumtoxinA (NeuBoNT-A), Letibotulinumtoxin A, botulinum toxin E (rBoNT-E), Innotox, and QM-1114 (Galderma) are being studied in clinical trials for different conditions. DaxiBoNT-A, NeuBoNT-A, and rBoNT-E are in clinical trials for potential indication in patients presenting with dystonia [67,73] (see Table 3). In practice, while being considered as therapies for disorders related to dystonia, there is critical lack of data regarding off-label uses. Additional clinical trials are required to expand the therapeutic applications of BoNT toward less conventional clinical applications. Considering the diversification of the new BoNT formulations currently in development, the therapeutic applications will certainly grow significantly in the near future [74].

For those reasons, practice guidelines were published by the American Academy of Neurology (AAN) in 2016 regarding the injection of botulinum toxin (BoNT) in the treatment of blepharospasm, cervical dystonia, adult spasticity, and headache, and were applicable to the four commercially available botulinum toxin formulations OnabotulinumtoxinA (ONA), AbobotulinumtoxinA (ABO), IncobotulinumtoxinA (INCO), and RimabotulinumtoxinB (RIMA). Guidance related to BoNT treatment was also included in the guidelines issued by the European Federation of the Neurological Societies (EFNS) focused on the treatment of primary dystonia [75].

While BoNT uses in blepharospasm and cervical dystonia are FDA-approved, BoNT is administered routinely for “off-label” presentations including, spasmodic dysphonia, oromandibular dystonia, truncal dystonia, limb dystonia, and tardive dystonia.

**Table 2:** Different formulations of BoNT-based therapies.

	Abo-Botulinum-Toxin A (Dysport^®^)	Inco-Botulinum-Toxin A (Xeomin^®^)	Ona-Botulinum-Toxin A (Botox^®^)	Rima-Botulinum-Toxin B (Myobloc^®^/Neurobloc^®^)	Pra-Botulinum-Toxin A (Nabota^®^)	Leti-Botulinum Toxin A (Botulax^®^)	Daxibotulinum-Toxin A (Daxxify^®^)
Toxinotype	A1	A1	A1	B	A1	A1	A1
Strain	Hall	Hall	Hall	Bean	Hall	CBFC26	Hall
Complex Size	>500 kD	150 kD	900 kD	700 kD	900 kD	900 kD	150 kD
Excipients	HSA (125 µg) Lactose	HSA (1 mg) Sucrose	HSA (500 µg) Sodium chloride	HSA (500 µg/mL) Sodium succinate Sodium chloride	HSA (500 µg/mL) Sodium Chloride 0.9 mg	HSA (250 µg) Sodium chloride 0.9 mg	RTP004 peptide 11.7 µg, L-histidine (0.14 mg), L-histidine-HCl monohydrate (0.65 mg), polysorbate 20 (0.1 mg), Trehalose dihydrate (36 mg).
Solubilization	Saline solution	Saline solution	Saline solution	N/A	Saline solution	Saline solution	
pH	7	7	7	5.6			5.5
Units/vial	300, 500	100, 200	100, 200	2500, 5000, 10,000	100	100, 200	50, 100
Shelf life (Months)	24	36	36	24		36	24
Protein content (ng/vial)	4.35	0.6	5	25, 50, 100			

Adapted from [71,72,76].

**Table 3:** Some recent BoNT formulations in clinical trials.

Name	Other Name	Toxinotype	Indications	Status	Origin
Prabotulinumtoxin A	ABP-450	BoNT/A1	Migraine, cervical distonia	Phase 2	AEON Biopharma, Irvine, CA, USA
Neubotulinumtoxin A	none	BoNT/A1	Primary axillary hyperhidrosis, cervical distonia	Phase 3	Medytox, Inc., Seoul, Republic of Korea
Letibotulinumtoxin A	none	BoNT/A1	Glabellar Lines	Phase 3	Hugel Inc., Seoul, Republic of Korea
Relabotulinumtoxin A	QM-1114	BoNT/A1	Glabellar lines and lateral canthal lines	Phase 3	Galderma, Courbevoie, France
Nivobotulinumtoxin A	Innotox	BoNT/A1	Glabellar Lines	Approved in Korea	Medytox, Inc., Seoul, Republic of Korea
Trenibotulinumtoxin E	none	BoNT/E	Glabellar Lines	Phase 3	Allergan Aesthetics, Irvine, CA, USA
A2NTX	none	BoNT/A2	Cervical distonia	Phase 1	Shionogi Pharma, Osaka, Osaka, Japan
IPN10200	none	Engineered A/B	Adult upper limb spasticity; upper facial lines	Phase 2	Ipsen Pharma, Paris, France
Gemibotulinumtoxin A	none	BoNT/A1	Post-operative atrial fibrillation	Phase 2	AbbVie, North Chicago, IL, USA
Daxibotulinumtoxin A	none	BoNT/A1	Adult upper limb spasticity; migraine	Phase 2	Revance, Nashville, TN, USA

## 3. An Expanding Range of Indications

In addition to the conventional indications as described above, BoNT-derived formulations are benefiting a much wider range of patients suffering from diverse disorders. Some of the “off-label” applications are usually approved by the regulators under emergency indication protocols, while the neurogenic bladder indication is already fully approved by the FDA and EMEA. 

### 3.1. Urology

Over the last 20 years, ONA and ABO have been extensively studied on Urological pathologies, first in patients suffering from various neurological disorders leading to neurogenic detrusor overactivity, then later in overactive bladder, and finally in painful bladder syndrome/interstitial cystitis (PBS/IC) patients.

Initially, the rationale for using botulinum toxin in urology practice was based on their strong inhibition on motor effects, through blockade of cholinergic neurotransmission in the detrusor. However, a huge number of preclinical studies in rodents suggested that the positive effects in reducing bladder contractility and micturition reflex could be mainly due to an effect on sensory pathways, through the inhibition of some neurotransmitters (i.e., nitric oxide, substance P, and CGRP) from the afferent nerve terminals. This hypothesis is strongly supported by the fact that antimuscarinics have limited efficacy in the majority of patients suffering from urinary bladder dysfunctions. However, most preclinical studies remain to be interpreted with caution considering the high doses used to observe positive effects on bladder contractility in vivo [77]. 

#### 3.1.1. Neurogenic Detrusor Overactivity (NDO)

Currently, only ONA is approved for the treatment of NDO [69]. NDO could be a consequence of neurological diseases like stroke, spinal cord injury (SCI), and Parkinson’s Disease, in addition to multiple sclerosis or transverse myelitis. NDO is characterised by several dysfunctions of the autonomic control of the urinary bladder and urethra because the signalling between the CNS and lower urinary tract is impaired. Urethral-sphincter dyssynergia induces a great increase in urinary bladder pressures and can lead to vesico-ureteral reflux and hydronephrosis. 

Recently, the results of two phase III randomised studies (CONTENT1 and CONTENT2) in patients with NDO treated with intra-detrusor injections of ABO 600 U or 800 U vs. placebo were published. ABO was well tolerated and significantly improved continence and bladder function, and QoL, in patients with SCI or MS with NDO [78]. These results could lead, in the near future, to the approval of ABO for NDO patients. 

A randomised, double-blind, non-inferiority clinical study was performed on the efficacy and tolerability of INCO vs. ONA intra-detrusor injections in patients with refractory NDO performing intermittent catheterization. The authors concluded that INCO was not inferior to ONA in improving clinical and urodynamic findings in the short-term follow-up, with comparable adverse effects [79]. 

Resistance to BTX-A is a significant problem in patients with NDO. A loss of efficacy over time has been described using ex vivo bladder model highlighting the need for a better understanding of the underlying mechanisms of BoNT actions [80]. Moreover, in a very limited study in four patients, INCO (which is free from complexing proteins) showed a good therapeutic response in one out of four patients, the only one not resistant to ONA in the extensor digitorum brevis (EBD) test [81]. The authors suggested that resistance could depend on antibodies working against the complex protein of ABO and ONA rather than from antibodies against the neurotoxin itself, as previously hypothesised. Nevertheless, the binding of antibodies to the accessory proteins does not automatically translate into the inhibition of the toxin’s effects, and the presence of antibodies does not automatically impact upon patient non-response to treatment [82]. Rather than a specific antibody response, the physiology of compensatory mechanisms should be further explored to explain the resistance to BoNT injections in NDO patients.

#### 3.1.2. Overactive Bladder

Today, only ONA is approved by FDA for the treatment of OAB. This condition affects a large percentage (between 10 and 20%) of the general population, and almost a third of these patients are affected by urinary incontinence, which severely impacts their quality of life. Anticholinergics are the most used pharmacological class of oral drugs, but their efficacy is limited and since they have numerous side effects and very high discontinuation rates are observed worldwide.

In the first large phase 3, placebo-controlled trial (EMBARK Study) of ONA in patients with overactive bladder and urinary incontinence inadequately managed with anticholinergics, ONA (100 U) significantly decreased the daily frequency of urinary incontinence episodes vs. the placebo. Moreover, 23% vs. 6% of patients became completely continent [83]. Urinary tract infection was the most common adverse event, followed by urinary retention. 

These results were confirmed by a study on Japanese patients, inadequately managed with anticholinergics and/or β3-adrenergic receptor agonists and injected with one dose of ONA at 100 U [84].

### 3.2. Off-Label Uses

#### 3.2.1. Interstitial Cystitis/Bladder Pain Syndrome (IC/BPS) 

This pathology is more common than previously reported [85]. The aetiology is unknown, but curiously, 90% of patients are women. Ulcer-type and non-ulcer-type IC/BPS are considered different disease entities. In a clinical study, it was found that repeated intravesical ONA injections provided some benefits in half of the patients with non-ulcer-type IC/BPS, but did not have any effect on patients with more severe disease, like ulcer-type IC/BPS [86].

In a recent phase II study on women, ONA 100 U was administered as 10 trigonal injections. The proportion of patients who achieved a 50% or greater reduction in the pain visual analogue scale was 60% for ONA vs. 22% for placebo, suggesting a significant therapeutic effect and good safety [87].

In conclusion, ONA is very effective in treating NDO and OAB at a dose of 100 U, since doses at 200 or 300 U are associated with more frequent side effect, mainly urinary tract infections and urinary retention, requiring urinary bladder catheterization. 

However, there is still a need to increase the efficacy and duration of the therapeutic effects of botulinum toxin in patients suffering from NDO, OAB, and IC/BPS and, in the meantime, to reduce side effects. Moreover, since a significant drawback is represented by toxin administration, i.e., detrusor injections performed at hospital under anaesthesia, some companies are developing new formulations of botulinum toxins to be administered through a catheter as an intravesical solution [88].

#### 3.2.2. Female Sexual Dysfunctions

There is growing evidence suggesting that BoNT is a safe and efficacious treatment option for female patients suffering from various sexual and genitourinary disorders, like dyspareunia, vaginismus, vestibulodynia, and persistent genital arousal disorder. In those patients, the BoNT is deemed to act by inhibiting the release of inflammatory neuropeptides CGRP and substance P and by inhibiting the acetylcholine release at the neuromuscular junction leading to reduced resting muscle tone. However, extensive research is still required to precisely identify the exact mechanisms in such disorders and to optimize the dose injected as well as the injection techniques [89]. In conclusion, more randomised controlled trial data regarding its long-term safety and efficacy are necessary to support these indications [90].

#### 3.2.3. Erectile Dysfunctions

The first indication of the beneficial effects of BoNT in erectile dysfunctions (ED) were evidenced during human and animal studies conducted by two different groups of investigators showing pro-erectile effects by an intra-cavernous injection of BoNT-A. The pro-erectile function could be due to cavernous smooth muscle relaxation by inhibition of noradrenaline release from the adrenergic neurons acting on the cavernous smooth muscle. In a clinical trial, an increase of 5 to 10% in erectile score in patients receiving BoNT was described [91,92]. Guliano et al. evidenced the role of sympathetic hyperactivity in ED which, therefore, can benefit from BoNT injections [93,94,95,96]. The erection mechanism implies a complex and delicate co-ordinated equilibrium between neurological, vascular and tissular compartments. There is a need for randomised placebo-controlled trials to further investigate the complex mechanisms leading to improved ED, since reliable data about sympathetic overactivity are missing to date. In addition, the current intracavernosal injection method will require further optimisations to improve patient comfort. Nevertheless, neither prolonged erection nor priapism were observed during the aforementioned clinical studies, in line with a reassuring locoregional safety profile and appropriate dosage of BoNT injections.

#### 3.2.4. Acute Dysmenorrhoea and Pelvic Pain Syndrome

Severe forms of dysmenorrhoea represent a common complaint in women and with major impact on their quality of life, fertility, productivity, and healthcare utilisation. Severe dysmenorrhea-limiting activity occurs in 16–29% of women and 5–7% have acute dysmenorrhoea [97]. The pelvic pain syndrome from uterine origin (painful uterine syndrome: PUS) is very frequently associated with severe dysmenorrhea syndrome along with culpability and hysterisation. Several magnetic resonance studies (cine-MRI) confirmed the significant increase in uterine myometrial hypercontractility in dysmenorrhoeic patients [98]. The PUS is the result of hypersensitivity and hypercontractility involving the pain regulation with exacerbated nociception involving substance P and leading to a decreased sensitivity threshold. Such sensibilisation mechanisms suggests considering the use of Botulinum Toxin (BTX) injections for treating acute dysmenorrhea and PUS. BTX injections have previously been recognised to be effective in patients with an overactive bladder, while BTX’s effectiveness is somewhat less evident in PUS. Nevertheless, its efficiency in PUS has been clearly confirmed in randomised controlled studies or meta-analyses [99,100,101].

#### 3.2.5. Chronic Pain

BoNTs are classically described as inhibiting the neurotransmitter release through exocytosis blockade. However, neuronal hyperexcitability driven by ionic currents has also been shown to be blocked by BoNT, demonstrating that BoNT actions are not limited to SNARE proteins cleavage [102]. The injection of BoNTs produced the inhibition of neurogenic inflammation in different pathologies like phantom pain and neuropathic neuralgia. In their clinical study of BOTNEP, Ranoux and coworkers established the beneficial effect of intradermal injection of BoNT in allodynia by improving the pain threshold [103,104]. However, the experimental data remain difficult to translate from healthy volunteers exposed to acute pain stimuli to sustained pathological pain since pain is greater in chronic pathological pain. Moreover, a possible central action of BoNT/A cannot be excluded since relief is obtained in the long term after one single injection. The elucidation of the mechanism of action is still required in the effects of BoNT/A in neuropathic pain. In addition, the topical application of BoNTs is considered in the elderly patients to avoid iatrogenia. In the future, toxin subtypes that would selectively target nociceptives fibres as well as cutaneous delivery techniques should be considered to improve the safety profile of BoNTs.

#### 3.2.6. Trigeminal Neuralgia

The application of BoNT in trigeminal neuralgia was evidenced by Zhang and coworkers using intradermal injections [105]. Nevertheless, considering the local side effects, experimental studies on new delivery formulations are required to optimise this indication since BoNT injections are also performed at specific trigger-points [104]. The clinical efficacy of BTX-A in trigeminal neuralgia is due to the inhibition of the release of inflammatory mediators and peripheral neurotransmitters from sensory nerves [106,107].

#### 3.2.7. Low Back Pain, Sciatica, and Pyalgia

BoNT injections are beneficial in lombalgia, pyalgia, myofascial pain, and sciatica. Stretching sessions are still useful before BoNT injections as well as massage of the painful site. Clinicians face the challenges of complicated diagnosis and lack of knowledge of BoNT pharmacology. However, a recommended consensus dosage of 100 units divided in 2 to 3 muscle points was agreed [108]. Although BoNT efficiency is recognised, several questions remain to clarify for this indication such as the localisation and dose regimen optimisation.

#### 3.2.8. Arthropathy

The intra-articular administration of BoNT/A for pain relief in musculoskeletal disorders has stirred great interest among patients suffering from long-term chronic pain. While BoNT was historically used in humans to reduce muscle spasticity, recent preclinical studies show an intrinsic intra-articular antinociceptive effect during BoNT treatment [109,110,111]. The pharmacodynamics behind pain modulation remain unclear since pain in arthropathy involves both nociceptive, neuropathic complex mechanisms, and abnormal excitability in peripheral and central pain pathways. BoNT is deemed to act as a “Swiss knife” by locally suppressing the neurotransmitter release leading to reduced peripheral sensitisation as well as decreased central sensitisation [112]. BoNT has an inhibitory role on the release of critical neuromediators involved in nociception, including substance P, calcitonin gene-related peptide, and glutamate. 

Arthropathy is usually treated with analgesics, corticoids, and anti-inflammatory drugs as well as hyaluronic acid; however, these drugs show a modest effect along with side effects. In this indication, the action of BoNTs can complete the therapeutic arsenal. To date, 14 randomised clinical trials have confirmed the analgesic effects of BoNT in this indication. The expected side effect consisting of possible reduced motor activity and lasting almost 8 weeks has been accepted by patients who all regularly return to the clinic to receive a new dose of BoNT because of its efficacy [113,114].

#### 3.2.9. Benefits of BoNT Injections in Cancer Therapies

More recently, many studies have raised awareness on the benefit of BoNT injections in painful symptoms associated with cancer and resulting from the direct pressure generated by the tumour or from chronic neuropathic pain after surgery or radiation. Nine studies among 10 clinical studies using a standardised scale to measure pain (VAS) demonstrated statistically significant improvement of the local pain at 4–8 weeks post-BoNT injection compared to baseline [115,116]. The use of BoNT injections can help to relieve pain in patients suffering from post radiation pain, hyperalgesia, and peripheral neuropathy after chemotherapy or postsurgical pain. For example, one single injection of BoNT can be sufficient in pectoralis muscles to avoid pain after a mastectomy. In the case of head and neck surgery, most clinicians agree that pain improves after injection of BoNT [117]. 

Patients with neuropathic pain due to spasm after a laryngectomy respond very well to 20 UI of Onabotulinum A for 10 days. Gastroparesis after oesophageal surgery is also greatly improved by 100 Units of Onabotulinum A. Nevertheless, more studies are still required to improve efficiency and safety of such injections. In addition to significant relief of pain, Onabotulinum A and Rimabotulinum B significantly reduce intense sweating of the face or excessive salivation post-surgery. The cessation of the hyperhydrosis due to parotid gland surgery, after BoNT injections is highly promising for the patient quality of life [118,119]. Parotid fistula healed rapidly after BoNT injections, while no serious adverse events were observed [120]. Chemotherapy-induced Raynaud syndrome is also improved after injection of BoNT in the hand. Tingling pain, spasm of the hand, and spasm of small arterioles are reduced, which can avoid ulcer and gangrene risk [121].

In parallel to clinical benefit in cancer patients, experimental studies show that BoNT exposure is efficient against cancer cells proliferation in culture and in vivo. Cellular apoptosis and the reduction in tumour size were evidenced after injections of BoNT into malignant tumour models associated with antiproliferative effects and increased caspase 3/7 activity [122,123,124]. Yet, the BoNT dose and exposure durations used in experimental studies are not directly transferable to the dosing regimen applied in human patients. Progress is being made to deliver BoNT to cells selectively (breast cancer cells in vitro) since early data suggest that the BoNT/A apoptotic activity may be selective for cancer cells, but additional studies are still required to assess the translational value of experimental in vivo or in vitro findings to human patients [125].

To summarise clinical and experimental findings, there is serious suggestion that BoNT can help cancer patients suffering from acute or chronic pain. Since the very origin of the pain comes from the muscle, from the neuropathic injury or the inflammation due to peripheral nerve entrapment, BoNT can bring an incredible benefit to the patient acting on each pathophysiological process. Botulinum toxin treatment offers two major benefits over classical pain remedies: BoNT injection effects last from 3 to 6 months and BoNTs are localised and safer than all potent analgesic agents. All the clinical studies performed recently in cancer patients after a surgery or a radiation therapy confirm the efficacy and safety of BoNTs on the pain associated with surgical and radiation therapies. BoNT is also very helpful in orofacial pain induced by chemotherapy.

Nevertheless, safety studies are required to guarantee safe dosing. Indeed, last year, a botulism outbreak occurred in Turkey following the injection of excessive BoNT doses in the stomach of patients [126]. Nevertheless, the potential benefit of BoNT is very promising because of the increasing incidence of cancer. 

Finally, it is worth noting that only two BoNT toxinotypes are currently approved for clinical use (BoNT/A and BoNT/B) despite the existence of at least eight different toxinotypes, BoNT A to H (or mosaic FA), thus suggesting a wider therapeutic potential [29]. Considering the increasing interest in therapeutic applications of BoNTs, it is becoming very attractive to enhance the properties of BoNTs by modulating its potency, eliminating its immunogenicity, extending the effects duration, developing fast acting formulations, or retargeting the toxin towards sensory neurons. Extending the duration of action of BoNT would benefit patients suffering from chronic conditions like muscle spasticity, overactive bladder or chronic migraine. The fast-acting formulations would be beneficial to deal with acute pain and also to expand the cosmetic uses. 

## 4. Exploration of Therapeutic Potential of BoNTs Toxinotypes or Subtypes

The rich diversity of BoNTs toxinotypes or subtypes serves as the foundation of new therapeutics. In addition, the considerable progress in sequencing is shedding light on the expanding BoNT superfamily as a natural repertoire available to support novel therapeutic options and the design of tailored toxins.

### 4.1. Variability between Toxinotypes

The pioneering studies on the structure of BoNTs by Lacy et al. described their moving modular shape “bringing the toxin molecule to life”. It became possible to grasp the correlation between structural particularities of each BoNT toxinotype and their functional diversity [127]. A key difference in subdomain organisation was evidenced in BoNT/E by Kumaran et al. in 2008, while BoNT/A and BoNT/B share a high degree of similarity. The catalytic and binding domains of the BoNT/E are arranged on the same side, which correlates well with the more rapid onset of action of BoNT/E. Those structural properties highlight that BoNTs toxinotypes do not share the same modular special organisation which greatly influences their effects [128,129]. 

In line with those differential effects and based on animal studies, the BoNT/B toxinotype was found to be less potent than anticipated in the clinic. The lower efficiency in humans was recently identified to be related to a residue difference within human synaptotagmin II (protein receptor for BoNT/B) [130]. This residue lies within the synaptotagmin II-binding cleft causing a lower affinity for BoNT/B than in other species. As a result, higher doses of BoNT/B need to be injected to achieve a similar efficacy than that achieved with BoNT/A [131]. This finding has prompted the design of optimised BoNT/B sequences binding to human synaptotagmin II with higher affinity and leading to enhanced efficacy. Although modified BoNT/B proteins show promising potential in the human neurons derived from induced pluripotent stem cells and transgenic humanised mouse models, the target doses need to match available BoNT/A1 products in order to claim a comparable efficacy and safety profile. The 4-fold difference in EC50 observed between rBoNT/A1 and rBoNT/B1 in the humanised mice contrasts with the 40-fold conversion factor used in the clinic between BoNT/A1 and BoNT/B1, but it still implies that the lower affinity of BoNT/B1 is not the only reason for its lower clinical efficacy. There are also large differences in the expression of human syt1 and syt2 between the different cell models that suggest only partial transferability to human tissues [132].

BoNT/A, BoNT/B, and BoNT/E cause neuromuscular paralysis for more than 4 months, ~2 months, or ≤4 weeks, respectively, when applied locally for the treatment of dystonia, illustrating the differences in neurotransmitter release blockade periods between different BoNT toxinotypes. The inhibition of transmitter release from cerebellar neurons by BoNT/A is far longer than with BoNT/B. Similarly, the BoNT/E and BoNT/F cause the short-lived blockade of transmitter release that coincides with the reappearance of intact SNAREs confirming their short half-lives of inhibition. The therapeutic potential of BoNT toxinotypes A1 to F1 were evaluated in ex vivo, in vitro, and in vivo assays. Except for toxinotype D1, all BoNT toxinotypes were evidenced as highly potent neurotoxins in rodent assays, in vivo. However, some intriguing dissimilarities between peripheral and central neurons suggest that further investigation is needed to clarify differences between toxinotypes in humans depending on the injection localization [133,134].

It is anticipated that the specificities of each BoNT toxinotype can lead to unique BoNT-based therapeutic formulations. In particular, toxinotypes F1 and C1 represent candidate therapies aimed at somatosensory system modulation [67]. The rapid refilling of synaptobrevin or SNAP-25 pools inside the cells explain the shorter effects of BoNT/F and BoNT/E, whereas the longevity of their respective proteases and cleavage products support the prolonged effects of BoNT/A, BoNT/B, and BoNT/C1 [133]. In addition, it was shown that the BoNT/A1 light chain’s longer lifetime is due to its ability to escape the cell degradation systems such as ubiquitination [134]. Conversely, the BoNT/E light chain is indeed ubiquitinated and driven towards the ubiquitin–proteasome system. This resistance to ubiquitination is due to the ability of BoNT/A light chain to recruit deubiquitinases that destroy the polyubiquitin chains acting within the ubiquitin–proteasome system. These differential durations of action can support the design of new BoNT formulations with differential pharmacokinetic profiles to fit different therapeutic needs [129,130,131,132,133,134,135,136]. Nevertheless, the differences in specific potency need to be interpreted with caution since they may be related to differences in toxin purity, toxin manufacture, or experimental conditions. Future, more detailed clinical studies on the different serotypes would help the elucidation of their pharmacokinetic profiles.

Assessing the differences in duration of action is paramount in clinical settings. This diversity between different BoNT toxinotypes provides to clinicians a unique opportunity to adapt the efficacy, duration of action, and antigenic potential of each BoNT formulation. BoNT/F was found to induce earlier sprouting and complete recovery faster than with classical formulations containing BoNT/A or BoNT/B. Electrophysiological studies can support physicians’ choices regarding BoNT formulations based on data obtained for each patient. The choices between different BoNT serotypes for the clinicians results from the analysis of preclinical and clinical studies, carefully assessing the relative efficacy, duration of action, safety, and antigenic potential of each serotype [136]. A new BoNT/A-B hybrid was designed, combining the high potency of BoNT/A with the high specificity of BoNT/B. Interestingly, in this study, the BoNT/A-B hybrid showed the same potency and duration than with BoNT/A-induced paresis in vivo, while the potency of the hybrid BoNT was 8-fold higher in the ex vivo hemidiaphragm assay. This combination will facilitate a dose reduction as well as a prolongation of the time interval between two administrations in autonomic disorders; however, differences between in vivo and ex vivo data show that the binding kinetics of BoNT/A-B hybrids differ between experimental settings [137].

In addition to modulation of the pharmacokinetic profile, the use of diverse toxinotypes can support new indications. Humans are not responsive to BoNT/DC targeting VAMP-2; however, the mosaic toxin BoNT-DC, but not BoNT-A, reduced melanin content in multiple melanocyte models, probably due to the cleavage of VAMP leading to the aberrant trafficking of tyrosinase and other cargo required for melanogenesis [138].

### 4.2. Variability between BoNT Subtypes

The duration of the effects of BoNT/A subtypes 1 to 5 was assessed in primary rat spinal neurons considering that the effects can reach 2–6 months in patients, in particular for BoNT/A. In this study, the intracellular activity of BoNT/A1, /A2, /A4, and /A5 could last for 10 months, whereas BoNT/A3 effects only lasted for 5 months. In the case of BoNT/E, the intracellular effect only lasted for 2–3 weeks. Those experimental findings demonstrate that the differential longevity of BoNT/A subtypes can be exploited to design long-lasting formulations [139].

The same authors revealed marked differences between the ten BoNT/A subtypes identified to date, consisting of differences in cell entry or proteases dynamics to differences in toxin potency in vivo and in vitro in the neuronal cell model. They confirmed that injections of BoNT/A1, /A2, and /A6 induced similar levels of paralysis as measured in the digital abduction paralysis model. Similarly, injections of BoNTs A1, A2, A6, and B1 resulted in a comparable pattern of paralysis using the Catwalk set-up. However, the effects of BoNT/B1 occurred one day later than with other BoNT/As [139]. The authors previously confirmed the comparable long-lasting effects of BoNT/A1, BoNT/A2, BoNT/A4, and BoNT/A5 subtypes in cultured primary neurons, while the BoNT/A3 effect was significantly shorter. In addition, a faster onset of paralysis was observed with a local injection of BoNT/A2 in vivo than with BoNT/A1, BoNT/A3, BoNT/A4, and BoNT/A5, as shown previously by different authors, while a faster recovery in motoneurons was observed with BoNT/A3. Currently, BoNT/A2 is being investigated in clinical trials in Japan, and A6 was also suggested as a potential new pharmaceutical. A clear benefit of the Catwalk model is the possible evaluation of the effects of BoNTs in a wide range of conditions like arthritis, spinal cord injury, Parkinson’s disease, multiple sclerosis, and stroke [140].

However, regarding BoNT variability, the functional properties of the various subtypes remain to be explored in additional in vitro or in vivo models. BoNT/A2 showed a faster onset than BoNT/A1 and a significantly higher potency in the in vivo and ex vivo models. In the same way, significant differences were evidenced between the paralytic effects of BoNT/B1 and BoNT/B2 ex vivo and in vivo [141,142]. Conversely, while causing different symptoms of intoxication in mice than with BoNT/A1, BoNT/A3 was found to be less potent and less effectively neutralised by anti-BoNT/A1 antibodies. These differences were suggested to originate from significant structural differences within the BoNT/A3 binding domain (Hc/A3) [143]. Likewise, the different mutations discovered in the BoNT/A3 and A4 binding domains may influence their binding dynamics and the different symptoms observed [144].

In addition to those findings, Tian and coworkers evidenced that while the nucleotide variability is uniformly distributed along the BoNT gene, the amino acid variability is not uniform within the full protein. The amino acid differences are focused along the light chain (LC) subdomain and the C-terminus of the receptor binding domain (HCC). Consequently, the LC region and the HCC region of BoNT are deemed to be the main source of BoNT differentiation. This finding explains the BoNT ability to bind to different receptors and cleave different substrates, thus targeting a wide variety of hosts [145]. Since the LC controls the catalytic properties and the duration of BoNT action, variations in the LC will directly impact the therapeutic properties of BoNT. Understanding such properties of the LC will support targeted applications of BoNT in human therapies [26]. 

This variability was confirmed in the study by Johnson and coworkers, who analysed two newly sequenced BoNT/A variants, Loch Maree (A3) and 657 Ba (A4), in comparison to A1 and A2. Combining sequence analysis, functional effects, molecular modelling and crystal structures of BoNT/A1 and the light chain of BoNT/A2, they concluded that the sequence differences within subtypes directly impact the isolation of efficient broad-spectrum antibody and small inhibitors. The significant differences between BoNT/A3 and BoNT/A4 in binding affinity and cleavage efficiency particularly affects their S1’ subsite recognition [146]. Further functional and modelling studies indicated that in rodent models, the disruption of HCc-V2C β-peptide and -glycan-N559 interactions mediates low BoNT/A4 potency, while in human motor neurons, the disruption of HCc-SV2C β-peptide alone mediates low BoNT/A4 potency due to species-specific variation at the SV2C site [147].

More recently, the progress made in the understanding of the lipid binding loop–interactions has supported the design of a more potent BoNT/B mutant. The mutant BoNT/B combines a stronger potency with lower blood diffusion than the original native BoNT/B. Therefore, this BoNT/B mutant combines higher safety with enhanced efficacy [148]. 

Considering the considerable progress made in BoNT variable structure elucidation, it becomes essential to translate recent structural findings into the design of more efficient and more specific therapies based on BoNTs protein models.

## 5. Modelling BoNTs for Non-Neuronal Targeting

While being one of the deadliest substances on earth, BoNT is used as a therapy in a variety of applications including dystonia or pain due to its exclusive ability to selectively target neuronal membranes. Using molecular engineering, this aptitude to select specific territories can now be exploited to target non-neuronal tissues while minimising systemic exposure [149]. Moreover, this precise targeting ability is essential to explore neuronal physiology or design restorative therapies. 

The initial approach by Dolly et al. consisted of engineering native BoNTs into inactive mutants (full-length BoNTs incorporating catalytically inactive LC/A or BoTIMs) and combining the inactive mutants with native LC/E domains. The resulting mosaic protein combined the persistence of LC/A with the fast onset of the LC/E action. This persistent cleavage of SNAP-25 may benefit patients with chronic pain syndromes or migraine [150,151]. In addition, the LC/E is more potent in the inhibition of CGRP release from sensory neurons, while the BoNT/A is more specifically targeting sensitive neurons, which confirms the benefit of combining the unique properties of each toxin component [152,153].

Studies were carried out to enhance the effect of LC/B on VAMP cleavage in vitro; however, the resulting BoNT/B mutant did exhibit a higher efficacy in in vitro assays (cell-based assays) or in vivo models [154]. The role of BoNT/C as a potential therapeutic toxin for synaptic transmission modulation was also evidenced by specific mutations enhancing the cleavage of syntaxin-1 [155,156]. Later, Elliott et al. isolated a newly engineered botulinum neurotoxin B combining improved binding ability with enhanced potency in preclinical models [132].

Compared to the modulation of the binding properties, the modulation of the translocation process remains challenging since the translocation dynamics remain a matter of debate. Mutated proteins conserve negatively charged residues in the LC and HN of BoNT/B on the basis that the protonation of these residues is required for the interaction of the toxin with the negatively charged membranes. Pirazzini and collaborators produced a triple mutant with a higher potency and faster onset time due to the protonation of the protein residues involved in the translocation of BoNTs. Nevertheless, BoNT protein engineering based on the translocation process remains challenging [157].

Their modular structure–function relationship makes BoNTs proteins more amenable candidates for retargeting to different territories. The fragment of BoNT/A composed of the LC and HN domains (LHN) unable to bind to neuronal cells has less toxicity, but the remanence of the translocation domain can still facilitate the formation of pores across the membrane under acidic conditions [158]. The crystal structure of LHN/A revealed a preserved structural relationship despite the absence of the HC domain, which makes the LHN fragment a common tool to deliver LC into cells that are not usually targeted by BoNTs in the nature [48,159]. The first studies employing this approach involved the use of an LHN/A preparation chemically coupled to lectin wheat germ agglutinin and nerve growth factor (NGF). The resulting conjugate was able to inhibit the release of noradrenaline from PC12 cells showing that the endopeptidase can be successfully delivered into a range of neuronal and non-neuronal cell types [160].

Based on the exclusive properties of the BoNTs, a new class of active proteins was created (TSIs: targeted secretion inhibitors; or TVEMP: targeted vesicular exocytosis-modulating protein) comprising three basic domains: (1) the LC domain of one selected BoNT toxinotype having the ability to cleave the SNARE proteins depending on the BoNT toxinotype, (2) the HN domain providing the intracellular translocation ability of the LC, and (3) the binding domain derived from BoNT or from a peptide or a protein interacting with a specific receptor on the target cell. This new approach exploits the endopeptidase domain to modulate the intracellular processes of the target cells by inhibiting their secretion mechanisms [5].

**Figure 1:**
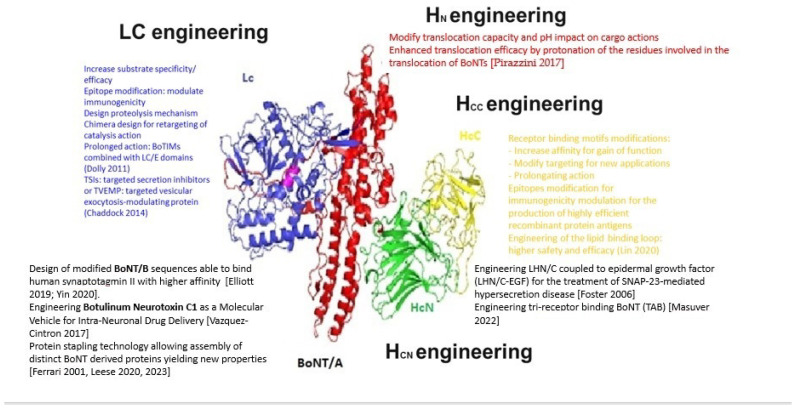
BoNT engineering options (adapted from references: [5,65,131,132,150,162,164,171,172,177]).

Figure 1 summarises the BoNT engineering opportunities.

The recombinant expression and purification of the LHN fragment of BoNT from *E. coli* allowed the combination of the LHN with a targeting entity. This technique can extend the cleavage activity to a variety of SNARE proteins opening new opportunities to target non-neuronal cell types that are not expressing SNAP-25 constitutively [161]. As an example, it is now possible to design new BoNTs to treat disorders related to SNAP-23-mediated hypersecretion based on the co-crystal structure, molecular dynamics, and mutagenesis findings. BoNT-derived fusion proteins are able to specifically target cell types involved in secretion mechanisms providing a basis to formulate new secretion inhibitors. A similar approach was used with the coupling of LHN/C to epidermal growth factor (LHN/C-EGF) to inhibit the release of mucin by pulmonary epithelial A549 cells in the treatment of asthma or chronic obstructive pulmonary disease [162].

The progress in understanding the structure–function relationship of BoNTs has permitted to overcome the SNAP-25 substrate limitations and extend the area where BoNTs may exercise its action [163,164,165]. The extension of BoNT substrate specificity has made possible to block the release of interleukin-8 (IL-8) and mucin from stimulated HeLa cell. This opportunity for a mutated LC part of BoNT/E to impact vesicle trafficking within non-neuronal tissue significantly expands the therapeutic applications of the BoNTs [166].

Another approach employs a protein-stapling technology to re-assemble BoNT/A from two separate fragments to generate a uniquely safe tool for neuroscience studies and new therapeutic applications. This BoNT-derived stapled protein chimera can reduce the mechanical hypersensitivity observed in a rat model of inflammatory pain and blocks the visual cortex neuronal activity but without inducing paralysis. Thus, this protein stapling technology allows the assembly of distinct BoNT fragments to yield new molecular properties without side effects [164]. 

Conversely, BoNT engineering may be used for the delivery of cargo or active compounds directly into neurons. Typically, the targeting of specific cells by biological toxins involves the delivery of their enzymatic moieties into the cytosol, but this delivery mode can be exploited through protein engineering to produce chimeric toxins. The *Clostridium botulinum* C2 binding and translocation domain was retargeted to neural cell populations by replacing its binding domain with a BoNT binding domain [167]. By using a comparable method, a bioengineered BoNT/C-vehicle was created with the ability to deliver therapeutic cargo into the neurons [168]. 

## 6. Future Approaches and Perspectives

Starting in 1822, when Justinus Kerner envisioned the therapeutic uses of the “fatty acid agents” that blocked the parasympathetic drive in animals, BoNT was isolated and purified at the beginning of the 20th century. Nevertheless, BoNT’s therapeutic potential was evidenced in strabismus near the end of the 20th century. While the molecular structure and modular composition of BoNT have been fully elucidated, the number of therapeutic indications are constantly rising and thus expanding their range from muscle spasticity, hyperhidrosis, migraine, chronic pain, and cosmetic applications to treatments for overactive bladder, erectile dysfunction, arthropathy, and cancer.

The engineering of BoNTs was inspired by its modular composition to design more potent, less toxic agents or to retarget the toxin at non-neuronal cells. Nevertheless, additional studies are required exploiting the engineering of the BoNTs to develop suitable therapies to treat acute or chronic pain. It is important to note that in vivo studies are implemented in healthy animals, which can be inappropriate in predicting clinical efficacy in spastic patients since major histopathological alterations are affecting the reactivity of spastic muscles [132]. Currently at the experimental step, the use of engineered BoNTs is becoming highly desirable for their neuroprotective effects, the influence on neuronal burgeoning, or on hormone secretion disorders. For example, a CGRP receptor antagonist delivered BoNT/D protease into sensory neurons and was found to inhibit K+-evoked substance P release. Since cytokines and neuropeptides are major regulators of inflammation and pain, blocking their release highlights the bio-therapeutic potential of engineered BoNTs in numerous chronic diseases [169].

In parallel to molecular engineering, studies on alternative existing toxinotypes evidenced the local and long-lasting paralysis effect of BoNT/CD comparable to that induced by BoNT/A but more efficient than BoNT/C. BoNT/CD was described as a potential replacement candidate to BoNT/A or BoNT/B in therapy or in cosmetic applications in the case of patients developing immunity [170].

In addition, a protein stapling technology was developed to combine two identical binding domains of tetanus and botulinum type D neurotoxins that enhanced intracellular delivery of molecules into neurons. The duplication of the binding parts of tetanus or botulinum neurotoxins allowed the production of large therapeutic enzymes penetrating neurons with higher efficiency [171]. The same authors developed a new isopeptide conjugation system to produce functional botulinum neuronal modulators using the two non-paralytic parts of the protein. This technique represents a safer approach for manufacturing therapeutic botulinum molecules. The elongated el-iBoNT molecule exhibited reduced paralytic ability compared to the non-elongated version and effectively alleviated nerve injury-induced pain in rats, an important improvement over previous stapled botulinum molecules [172]. Those recombinant forms of BoNTs show the potential to be targeted to specific neurons and by removing the paralytic moiety of the toxin, this technique will automatically enhance safety and improve the benefit/risk ratio in therapy.

Regarding the exploitation of differences between toxinotypes, engineering will allow the improved binding of BoNT/E to SV2c by building a BoNT/A-like binding pocket within BoNT/E. The modifications of native BoNT/E provide novel avenues for fast acting BoNT/E-based products [173]. A new recombinant BoNT/E has recently been assessed in a first clinical study showing a faster onset of action, greater peak, and shorter duration of effect versus abobotulinumtoxinA. This rBoNT-E demonstrated a good safety profile and will allow unmet therapeutic and aesthetic patient needs to be addressed [174]. The faster onset of action and shorter duration of effect was already confirmed in vivo in the mouse DAS assay; however, this study employed the native form of BoNT/E [175].

The specificity of BoNT/A resides in its high potency and specific binding but with a lag phase. BoNT/A can now be combined to fast acting inhibitors to gain fast action in parallel to long-term effects. A combination of conotoxin with BoNT/A has been shown to accelerate and potentiate the effects of BoNT/A [176]. In parallel to faster action, a new tri-receptor binding BoNT (TAB) has been designed to improve native BoNT pharmacological profile by optimisation of the binding properties [177].

Studies on the effects of BoNTs at territories remote from the injection site will support the development of BoNT therapies specific to certain organs. Currently, the pharmaceutical development of liquid or slow-release BoNT formulations for transdermal, trans-urothelial, and transepithelial delivery remains highly challenging. Progress in the formulation and delivery techniques in parallel to more sensitive analytical techniques will be paramount to deliver efficient next-generation BoNT clinical products [48]. Injection-free delivery has been tested using liposomal BoNT (lipotoxin). This hydrogel formulation (Theracoat TC-3) allowed the slow release of BoNT, but its efficacy remained elusive showing that considerable work is needed to develop efficient “injection-free” formulations [178]. In addition to liposome formulations, microneedle devices (DMNPs) were prepared to test the functionality of the BoNT/A/DMNPs on the hyperhidrosis mouse footpad, showing drastically reduced sweat gland activity. Those innovative results demonstrate that these devices such as microneedles (DMNPs) can be an effective and painless alternative to hypodermic injections when treating hyperhidrosis with BoNT/A [179,180].

Nevertheless, significant progress has been made regarding the increased duration of action with the DaxibotulinumtoxinA (Daxxify), a novel BoNTA product containing highly purified 150 kDa core neurotoxin formulated with a proprietary stabilising excipient peptide (RTP004) instead of human serum albumin. The positively charged RTP004 enhanced the binding of the neurotoxin to neuronal surfaces, which facilitates neurotoxin internalisation. However, its extended duration of action is also provided by the higher dose of BoNT/A contained in this formulation [61]. A new analgesic formulation (N-001) was engineered from several *C. botulinum* toxins and targeting sensory neurons resulting in pain relief lasting for 3 days. Those new results encourage further studies using N-001 as a potential analgesic for post-operative pain treatment [181].

Another important future aspect will consist in optogenetics approaches allowing the local activation of the toxin. This formulation of photoactivable BoNTs can be induced to control locally the neuronal transmission [182]. Beyond the activation of BoNTs through optogenetics, non-replicative viral vectors based on HSV-1 viral particles (Herpes simplex Virus type 1) can express the LC of BoNT/A, BoNT/B, BoNT/C, BoNT/D, BoNT/E, and BoNT/F after infecting embryonic and adult rat DRG neurons. The resulting transgenic BoNT LCs cleave SNARE proteins, thereby inhibiting the release of CGRP by embryonic rat sensory neurons. A vector-based strategy could provide a continuous production of the LC intracellularly, and thus avoid limitations arising from the need to repeat BoNT administrations and avoid potential resistance to the treatment [183].

To summarise, native BoNT/A or BoNT/B represent widely recognised, effective, and safe treatments option for a variety of disorders ranging from muscle spasticity and hyperhidrosis to chronic pain, as well as having had global success in the cosmetic industry. On the other hand, the optimisation of current formulations is becoming increasingly sought-after considering the wide potential of the natural BoNT repertoire as well as the many engineering opportunities lying within this modular protein. Furthermore, new types of formulations could make BoNT treatments more selective for unique territories while enhancing their local efficacy and reducing the risk of side effects. However, further clinical studies are needed to increase the safety margin in the new treatment indications as well as for the new types of formulations. Despite the high safety profile of present botulinum toxin formulations, the potential risks related to its intentional and inappropriate uses need to be thoroughly addressed in view of the expanding formulations and indications. The existing regulatory framework will require strengthening around the ever-increasing therapeutic indications. This move towards recombinant and local BoNT formulations (topic gels, lotions, and microneedle devices) needs to be backed up with comprehensive safety evaluations both in preclinical and clinical steps. On a positive note, the use of recombinant biological toxins represents a major advance towards perfect reliability in toxin production, toxin calibration, and potency quantification. Newly developed reference materials based on recombinant forms of BoNTs will serve as the basis for the evaluation of different detection methods and will be the benchmarks for the assessment of toxin potency between different laboratories [184].

## Data Availability

The original contributions presented in the study are included in the article, further inquiries can be directed to the corresponding author.

## References

[1] Erbguth F.J., Naumann M. (1999). Historical aspects of botulinum toxin: Justinus Kerner (1786–1862) and the “sausage poison”. Neurology.

[2] van Ermengem E. (1979). A New Anaerobic Bacillus and Its Relation to Botulism. Rev. Infect. Dis..

[3] Popoff M.R., Nikolay S. (2018). Botulinum toxins, Diversity, Mode of Action, Epidemiology of Botulism in France. Botulinum Toxin.

[4] Scott A.B., Magoon E.H., McNeer K.W., Stager D.R. (1989). Botulinum treatment of strabismus in children. Trans. Am. Ophthalmol. Soc..

[5] Chaddock J.A., Foster K.A. (2014). Future Developments: Engineering the Neurotoxin. Clinical Applications of Botulinum Neurotoxin.

[6] Mazuet C., Legeay C., Sautereau J., Ma L., Bouchier C., Bouvet P., Popoff M. (2016). Diversity of Group I and II Clostridium botulinum Strains from France Including Recently Identified Subtypes. Genome Biol. Evol..

[7] Aureli P., Fenicia L., Pasolini B., Gianfranceschi M., McCroskey L.M., Hatheway C.L. (1986). Two cases of type E infant botulism caused by neurotoxigenic Clostridium butyricum in Italy. J. Infect. Dis..

[8] Arnon S.S., Schechter R., Inglesby T.V., Henderson D.A., Bartlett J.G., Ascher M.S., Eitzen E., Fine A.D., Hauer J., Layton M. (2001). Botulinum toxin as a biological weapon: Medical and public health management. JAMA.

[9] Hatheway C.L. (1990). Toxigenic clostridia. Clin. Microbiol. Rev..

[10] Rossetto O., Seveso M., Caccin P., Schiavo G., Montecucco C. (2001). Tetanus and botulinum neurotoxins: Turning bad guys into good by research. Toxicon.

[11] Rigoni M., Caccin P., Johnson E.A., Montecucco C., Rossetto O. (2001). Site-directed mutagenesis identifies active-site residues of the light chain of botulinum neurotoxin type A. Biochem. Biophys. Res. Commun..

[12] Schiavo G., Matteoli M., Montecucco C. (2000). Neurotoxins affecting neuroexocytosis. Physiol. Rev..

[13] Peck M.W., Smith T.J., Anniballi F., Austin J.W., Bano L., Bradshaw M., Cuervo P., Cheng L.W., Derman Y., Dorner B. (2017). Historical Perspectives and Guidelines for Botulinum Neurotoxin Subtype Nomenclature. Toxins.

[14] Montecucco C., Schiavo G. (1995). Structure and function of tetanus and botulinum neurotoxins. Q. Rev. Biophys..

[15] Montal M. (2010). Botulinum neurotoxin: A marvel of protein design. Annu. Rev. Biochem..

[16] Fischer A., Montal M. (2013). Molecular dissection of botulinum neurotoxin reveals interdomain chaperone function. Toxicon.

[17] Muraro L., Tosatto S., Motterlini L., Rossetto O., Montecucco C. (2009). The N-terminal half of the receptor domain of botulinum neurotoxin A binds to microdomains of the plasma membrane. Biochem. Biophys. Res. Commun..

[18] Herreros J., Schiavo G. (2002). Lipid microdomains are involved in neurospecific binding and internalisation of clostridial neurotoxins. Int. J. Med. Microbiol..

[19] Lalli G., Herreros J., Osborne S.L., Montecucco C., Rossetto O., Schiavo G. (1999). Functional characterisation of tetanus and botulinum neurotoxins binding domains. J. Cell Sci..

[20] Collins M.D., East A.K. (1998). Phylogeny and taxonomy of the food-borne pathogen Clostridium botulinum and its neurotoxins. J. Appl. Microbiol..

[21] Gu S., Jin R. (2013). Assembly and function of the botulinum neurotoxin progenitor complex. Curr. Top. Microbiol. Immunol..

[22] Lee K., Zhong X., Gu S., Kruel A.M., Dorner M.B., Perry K., Rummel A., Dong M. (2014). and R. Jin Molecular basis for disruption of E-cadherin adhesion by botulinum neurotoxin A complex. Science.

[23] Fujinaga Y., Matsumura T., Jin Y., Takegahara Y., Sugawara Y. (2009). A novel function of botulinum toxin-associated proteins: HA proteins disrupt intestinal epithelial barrier to increase toxin absorption. Toxicon.

[24] Aoki K.R. (2001). Pharmacology and immunology of botulinum toxin serotypes. J. Neurol..

[25] Rossetto O., Pirazzini M., Montecucco C. (2014). Botulinum neurotoxins: Genetic, structural and mechanistic insights. Nat. Rev. Microbiol..

[26] Gardner A.P., Barbieri J.T. (2018). Light Chain Diversity among the Botulinum Neurotoxins. Toxins.

[27] Smith L.A., Rusnak J.M. (2007). Botulinum neurotoxin vaccines: Past, present, and future. Crit. Rev. Immunol..

[28] Peck M.W., Stringer S.C., Carter A.T. (2011). Clostridium botulinum in the post-genomic era. Food Microbiol..

[29] Barash J.R., Arnon S.S. (2014). A novel strain of Clostridium botulinum that produces type B and type H botulinum toxins. J. Infect. Dis..

[30] Dover N., Barash J.R., Hill K.K., Xie G., Arnon S.S. (2014). Molecular characterization of a novel botulinum neurotoxin type H gene. J. Infect. Dis..

[31] Maslanka S.E., Luquez C., Dykes J.K., Tepp W.H., Pier C.L., Pellett S., Raphael B., Kalb S.R., Barr J.R., Rao A. (2016). A Novel Botulinum Neurotoxin, Previously Reported as Serotype H, Has a Hybrid-Like Structure with Regions of Similarity to the Structures of Serotypes A and F and Is Neutralized with Serotype A Antitoxin. J. Infect. Dis..

[32] Zhang S., Masuyer G., Zhang J., Shen Y., Lundin D., Henriksson L., Miyashita S., Martínez-Carranza M., Dong M. (2017). and P. Stenmark. Identification and characterization of a novel botulinum neurotoxin. Nat. Commun..

[33] Zhang S., Lebreton F., Mansfield M.J., Miyashita S.I., Zhang J., Schwartzman J.A., Tao L., Masuyer G., Martínez-Carranza M., Stenmark P. (2018). Identification of a Botulinum Neurotoxin-like Toxin in a Commensal Strain of Enterococcus faecium. Cell Host Microbe.

[34] Contreras E., Masuyer G., Qureshi N., Chawla S., Dhillon H.S., Lee H.L., Chen J., Stenmark P., Gill S. (2019). A neurotoxin that specifically targets Anopheles mosquitoes. Nat. Commun..

[35] Wei X.W.T., Lobb B., Mansfield M., Zhen W., Tan H., Wu Z., Pellett S., Dong M., Doxey A.C. (2022). Identification of divergent botulinum neurotoxin homologs in *Paeniclostridium ghonii*. bioRxiv.

[36] Guo J., Pan X., Zhao Y., Chen S. (2013). Engineering Clostridia Neurotoxins with elevated catalytic activity. Toxicon.

[37] Masuyer G., Zhang S., Barkho S., Shen Y., Henriksson L., Kosenina S., Dong M., Stenmark P. (2018). Structural characterisation of the catalytic domain of botulinum neurotoxin X-high activity and unique substrate specificity. Sci. Rep..

[38] Gregg B.M., Matsumura T., Wentz T.G., Tepp W.H., Bradshaw M., Stenmark P., Johnson E.A., Fujinaga Y., Pellett S. (2024). Botulinum neurotoxin X lacks potency in mice and in human neurons. mBio.

[39] Zornetta I., Azarnia Tehran D., Arrigoni G., Anniballi F., Bano L., Leka O., Zanotti G., Binz T., Montecucco C. (2016). The first non Clostridial botulinum-like toxin cleaves VAMP within the juxtamembrane domain. Sci. Rep..

[40] Smith T.J., Schill K.M., Williamson C.H.D. (2023). Navigating the Complexities Involving the Identification of Botulinum Neurotoxins (BoNTs) and the Taxonomy of BoNT-Producing Clostridia. Toxins.

[41] Schantz E.J., Johnson E.A. (1992). Properties and use of botulinum toxin and other microbial neurotoxins in medicine. Microbiol. Rev..

[42] Scott A.B., Honeychurch D., Brin M.F. (2023). Early development history of Botox (onabotulinumtoxinA). Medicine.

[43] Ting P.T., Freiman A. (2004). The story of Clostridium botulinum: From food poisoning to Botox. Clin. Med..

[44] Dressler D. (2013). Botulinum toxin therapy: Its use for neurological disorders of the autonomic nervous system. J. Neurol..

[45] Dressler D. (2016). Botulinum toxin drugs: Brief history and outlook. J. Neural Transm..

[46] Jankovic J. (1998). Medical therapy and botulinum toxin in dystonia. Adv. Neurol..

[47] Jankovic J., Brin M.F. (1991). Therapeutic uses of botulinum toxin. N. Engl. J. Med..

[48] Fonfria E., Maignel J., Lezmi S., Martin V., Splevins A., Shubber, SKalinichev M., Foster K., Picaut P., Krupp J. (2018). The Expanding Therapeutic Utility of Botulinum Neurotoxins. Toxins.

[49] Laing T.A., Laing M.E., O’Sullivan S.T. (2008). Botulinum toxin for treatment of glandular hypersecretory disorders. J. Plast. Reconstr. Aesthet. Surg..

[50] Kumar R., Dhaliwal H.P., Kukreja R.V., Singh B.R. (2016). The Botulinum Toxin as a Therapeutic Agent: Molecular Structure and Mechanism of Action in Motor and Sensory Systems. Semin. Neurol..

[51] Aurora S.K., Winner P., Freeman M.C., Spierings E.L., Heiring J.O., DeGryse R.E., VanDenburgh A., Nolan M.E., Turkel C. (2011). OnabotulinumtoxinA for treatment of chronic migraine: Pooled analyses of the 56-week PREEMPT clinical program. Headache.

[52] Kim H.J., Lee G.W., Kim M.J., Yang K.Y., Kim S.T., Bae Y.C., Ahn D.K. (2015). Antinociceptive Effects of Transcytosed Botulinum Neurotoxin Type A on Trigeminal Nociception in Rats. Korean J. Physiol. Pharmacol..

[53] Burstein R., Zhang X., Levy D., Aoki K.R., Brin M.F. (2014). Selective inhibition of meningeal nociceptors by botulinum neurotoxin type A: Therapeutic implications for migraine and other pains. Cephalalgia.

[54] Grando S.A., Zachary C.B. (2018). The non-neuronal and nonmuscular effects of botulinum toxin: An opportunity for a deadly molecule to treat disease in the skin and beyond. Br. J. Dermatol..

[55] Schlessinger J., Gilbert E., Cohen J.L., Kaufman J. (2017). New Uses of AbobotulinumtoxinA in Aesthetics. Aesthet. Surg. J..

[56] Jung B.H., Song S.H., Yoon S.J., Koo J.H., Yoo K.Y. (2022). The Effect of Botulinum Toxin on Hair Follicle Cell Regeneration Under Continuous Stress Conditions: A Pilot Animal Study. Neurotox. Res..

[57] Hanchanale V.S., Rao A.R., Martin F.L., Matanhelia S.S. (2010). The unusual history and the urological applications of botulinum neurotoxin. Urol. Int..

[58] Anandan C., Jankovic J. (2021). Botulinum Toxin in Movement Disorders: An Update. Toxins.

[59] Scaglione F. (2016). Conversion Ratio between Botox^®^, Dysport^®^, and Xeomin^®^ in Clinical Practice. Toxins.

[60] DAXXIFY® Becomes First Facial Injectable to be Named to TIME’s Best Inventions. https://www.businesswire.com/news/home/20231026772907/en/DAXXIFY%C2%AE-Becomes-First-Facial-Injectable-to-be-Named-to-TIME%E2%80%99s-Best-Inventions.

[61] Solish N., Carruthers J., Kaufman J., Rubio R.G., Gross T.M., Gallagher C.J. (2021). Overview of DaxibotulinumtoxinA for Injection: A Novel Formulation of Botulinum Toxin Type A. Drugs.

[62] Dressler D. (2020). Therapeutically relevant features of botulinum toxin drugs. Toxicon.

[63] Gary D. (2017). Monheit, Andy Pickett AbobotulinumtoxinA: A 25-Year History. Aesthet. Surg. J..

[64] Fernández-Salas E., Wang J., Molina Y., Nelson J.B., Jacky B.P., Aoki K.R. (2012). Botulinum neurotoxin serotype A specific cell-based potency assay to replace the mouse bioassay. PLoS ONE.

[65] Rasetti-Escargueil C., Lemichez E., Popoff M.R. (2018). Variability of Botulinum Toxins: Challenges and Opportunities for the Future. Toxins.

[66] Dressler D., Johnson E.A. (2022). Botulinum toxin therapy: Past, present and future developments. J. Neural Transm..

[67] Choudhury S., Baker M.R., Chatterjee S., Kumar H. (2021). Botulinum Toxin: An Update on Pharmacology and Newer Products in Development. Toxins.

[68] Inukai Y. (1963). Role of proteolytic enzyme in toxin production by clostridium botulinum type A. Jpn. J. Vet. Res..

[69] Jones R.G., Liu Y., Halls C., Thorpe S.J., Longstaff C., Matejtschuk P., Sesardic D. (2011). Release of proteolytic activity following reduction in therapeutic human serum albumin containing products: Detection with a new neoepitope endopeptidase immunoassay. J. Pharm. Biomed. Anal..

[70] Brin M.F., James C., Maltman J. (2014). Botulinum toxin type A products are not interchangeable: A review of the evidence. Biologics.

[71] Donald S., Elliott M., Gray B., Hornby F., Lewandowska A., Marlin S., Favre-Guilmard C., Périer C., Cornet S., Kalinichev M. (2018). A comparison of biological activity of commercially available purified native botulinum neurotoxin serotypes A1 to F1 in vitro, ex vivo, and in vivo. Pharmacol. Res. Perspect..

[72] Klein A.W., Carruthers A., Fagien S., Lowe N.J. (2008). Comparisons among botulinum toxins: An evidence-based review. Plast. Reconstr. Surg..

[73] Takeuchi T., Okuno T., Miyashiro A., Kohda T., Miyamoto R., Izumi Y., Kozaki S., Kaji R. (2021). Clinical Safety and Tolerability of A2NTX, a Novel Low-Molecular-Weight Neurotoxin Derived from Botulinum Neurotoxin Subtype A2, in Comparison with Subtype A1 Toxins. Toxins.

[74] Spiegel L.L., Ostrem J.L., Bledsoe I.O. (2020). FDA Approvals and Consensus Guidelines for Botulinum Toxins in the Treatment of Dystonia. Toxins.

[75] Albanese A., Abbruzzese G., Dressler D., Duzynski W., Khatkova S., Marti M.J., Mir P., Montecucco C., Moro E., Pinter M. (2015). Practical guidance for CD management involving treatment of botulinum toxin: A consensus statement. J. Neurol..

[76] FDA (2023). BOTOX (onabotulinumtoxinA) Label. Allergan, Inc. U.S. U.S. Food and Drug Administration Website. https://www.accessdata.fda.gov/Drugsatfda_docs/Label/2011/103000s5236lbl.Pdf.

[77] Dieter A.A., Wu J.M., Siddiqui N.Y., Degoski D.J., Brooks J.M., Dolber P.C., Fraser M.O. (2016). Characterizing the Bladder’s Response to Onabotulinum Toxin Type A Using a Rat Model. Female Pelvic. Med. Reconstr. Surg..

[78] Denys P., Joussain C. (2023). Intradetrusor botulinum toxin as the first-line treatment for neurogenic detrusor overactivity: Pro. Prog. Urol..

[79] Giannantoni A., Gubbiotti M., Rubilotta E., Balzarro M., Antonelli A., Bini V. (2022). IncobotulinumtoxinA versus onabotulinumtoxinA intradetrusor injections in patients with neurogenic detrusor overactivity incontinence: A double-blind, randomized, non-inferiority trial. Minerva Urol. Nephrol..

[80] Maignel J., Martin V., Assaly R., Vogt M.L., Retailleau K., Hornby F., Laugerotte A., Lezmi S., Denys P., Krupp J. (2022). BoNT/A1 Secondary Failure for the Treatment of Neurogenic Detrusor Overactivity: An Ex Vivo Functional Study. Toxins.

[81] Andretta E., Zuliani C., Cavallari F., Artuso G. (2013). Poster 670, 43rd Annual Meeting of the International Continence Society. Neurourol. Urodyn..

[82] Benecke R. (2012). Clinical relevance of botulinum toxin immunogenicity. BioDrugs.

[83] Nitti V.W., Dmochowski R., Herschorn S., Sand P., Thompson C., Nardo C., Yan X., Haag-Molkenteller C., EMBARK Study Group (2017). OnabotulinumtoxinA for the Treatment of Patients with Overactive Bladder and Urinary Incontinence: Results of a Phase 3, Randomized, Placebo Controlled Trial. J. Urol..

[84] Yokoyama O., Honda M., Yamanishi T., Sekiguchi Y., Fujii K., Nakayama T., Mogi T. (2020). OnabotulinumtoxinA (botulinum toxin type A) for the treatment of Japanese patients with overactive bladder and urinary incontinence: Results of single-dose treatment from a phase III, randomized, double-blind, placebo-controlled trial (interim analysis). Int. J. Urol..

[85] Leppilahti M., Sairanen J., Tammela T.L., Aaltomaa S., Lehtoranta K., Auvinen A. (2005). Finnish Interstitial Cystitis-Pelvic Pain Syndrome Study Group Prevalence of clinically confirmed interstitial cystitis in women: A population based study in Finland. J. Urol..

[86] Lee C.L., Kuo H.C. (2013). Intravesical botulinum toxin a injections do not benefit patients with ulcer type interstitial cystitis. Pain Physician.

[87] Pinto R.A., Costa D., Morgado A., Pereira P., Charrua A., Silva J., Cruz F. (2018). Intratrigonal OnabotulinumtoxinA Improves Bladder Symptoms and Quality of Life in Patients with Bladder Pain Syndrome/Interstitial Cystitis: A Pilot, Single Center, Randomized, Double-Blind, Placebo Controlled Trial. J. Urol..

[88] Chuang Y.C., Kuo H.C. (2017). A Prospective, Multicenter, Double-Blind, Randomized Trial of Bladder Instillation of Liposome Formulation OnabotulinumtoxinA for Interstitial Cystitis/Bladder Pain Syndrome. J. Urol..

[89] Dick B., Natale C., Reddy A., Akula K.P., Yousif A., Hellstrom W.J.G. (2021). Application of Botulinum Neurotoxin in Female Sexual and Genitourinary Dysfunction: A Review of Current Practices. Sex. Med. Rev..

[90] Reddy A.G., Dick B.P., Natale C., Akula K.P., Yousif A., Hellstrom W.J.G. (2021). Application of Botulinum Neurotoxin in Male Sexual Dysfunction: Where Are We Now?. Sex. Med. Rev..

[91] Ghanem H., Raheem A.A., AbdelRahman I.F.S., Johnson M., Abdel-Raheem T. (2018). Botulinum Neurotoxin and Its Potential Role in the Treatment of Erectile Dysfunction. Sex. Med. Rev..

[92] Ghanem H.M. (2017). Re: Botox for Erectile Dysfunction. J. Sex. Med..

[93] Giuliano F., Denys P., Joussain C. (2023). Safety and Effectiveness of Repeated Botulinum Toxin A Intracavernosal Injections in Men with Erectile Dysfunction Unresponsive to Approved Pharmacological Treatments: Real-World Observational Data. Toxins.

[94] Giuliano F., Denys P., Joussain C. (2022). Effectiveness and Safety of Intracavernosal IncobotulinumtoxinA (Xeomin^®^) 100 U as an Add-on Therapy to Standard Pharmacological Treatment for Difficult-to-Treat Erectile Dysfunction: A Case Series. Toxins.

[95] Giuliano F., Joussain C., Denys P. (2022). Long Term Effectiveness and Safety of Intracavernosal Botulinum Toxin A as an Add-on Therapy to Phosphosdiesterase Type 5 Inhibitors or Prostaglandin E1 Injections for Erectile Dysfunction. J. Sex. Med..

[96] Giuliano F., Joussain C., Denys P., Laurin M., Behr-Roussel D., Assaly R. (2022). Intracavernosal OnabotulinumtoxinA Exerts a Synergistic Pro-Erectile Effect When Combined With Sildenafil in Spontaneously Hypertensive Rats. J. Sex. Med..

[97] Ju H., Jones M., Mishra G. (2014). The prevalence and risk factors of dysmenorrhea. Epidemiol. Rev..

[98] Kataoka M., Togashi K., Kido A., Nakai A., Fujiwara T., Koyama T., Fujii S. (2005). Dysmenorrhea: Evaluation with cine-mode-display MR imaging--initial experience. Radiology.

[99] Bautrant E., Franke O., Amiel C., Bensousan T., Thiers-Bautrant D., Leveque C. (2021). Treatment of acute dysmenorrhoea and pelvic pain syndrome of uterine origin with myometrial botulinum toxin injections under hysteroscopy: A pilot study. J. Gynecol. Obstet. Hum. Reprod..

[100] Levesque A., Ploteau S., Michel F., Siproudhis L., Bautrant E., Eggermont J., Fujii S. (2021). Botulinum toxin infiltrations versus local anaesthetic infiltrations in pelvic floor myofascial pain: Multicentre, randomized, double-blind study. Ann. Phys. Rehabil. Med..

[101] Martial Kouame J., Leveque C., Siani C., Santos M., Delorme J., Franke O., Amiel C., Bensousan T., Thiers-Bautrant D., Bautrant E. (2023). Uterine botulinum toxin injections in severe dysmenorrhea, dyspareunia and chronic pelvic pain: Results on quality of life, pain level and medical consumption. Eur. J. Obstet. Gynecol. Reprod. Biol..

[102] Humeau Y., Doussau F., Grant N.J., Poulain B. (2000). How botulinum and tetanus neurotoxins block neurotransmitter release. Biochimie.

[103] Ranoux D., Attal N., Morain F., Bouhassira D. (2008). Botulinum toxin type A induces direct analgesic effects in chronic neuropathic pain. Ann. Neurol..

[104] Ranoux D., Levine R.A. (2022). Botulinum Toxin Can Abolish and/or Quiet Tinnitus Associated with Chronic Migraine: Serendipidous Observations. Int. Tinnitus J..

[105] Zhang H., Lian Y., Ma Y., Chen Y., He C., Xie N., Wu C. (2014). Two doses of botulinum toxin type A for the treatment of trigeminal neuralgia: Observation of therapeutic effect from a randomized, double-blind, placebo-controlled trial. J. Headache Pain.

[106] Rubis A., Juodzbalys G. (2020). The Use of Botulinum Toxin A in the Management of Trigeminal Neuralgia: A Systematic Literature Review. J. Oral Maxillofac. Res..

[107] Kayani A.M.A., Silva M.S., Jayasinghe M., Singhal M., Karnakoti S., Jain S., Jena R. (2022). Therapeutic Efficacy of Botulinum Toxin in Trigeminal Neuralgia. Cureus.

[108] Dupeyron A., Viollet E., Coroian F., Gagnard C., Renard D., Castelnovo G. (2015). Botulinum Toxin-A for treatment of Pisa syndrome: A new target muscle. Park. Relat. Disord..

[109] Munoz-Lora V.R.M., Dugonjic Okrosa A., Matak I., Del Bel Cury A.A., Kalinichev M., Lackovic Z. (2022). Antinociceptive Actions of Botulinum Toxin A1 on Immunogenic Hypersensitivity in Temporomandibular Joint of Rats. Toxins.

[110] Luvisetto S. (2022). Botulinum Neurotoxins beyond Neurons: Interplay with Glial Cells. Toxins.

[111] Luvisetto S. (2021). Botulinum Neurotoxins in Central Nervous System: An Overview from Animal Models to Human Therapy. Toxins.

[112] Anderson S., Krug H., Dorman C., McGarraugh P., Frizelle S., Mahowald M. (2010). Analgesic effects of intra-articular botulinum toxin Type B in a murine model of chronic degenerative knee arthritis pain. J. Pain Res..

[113] Nguyen C., Rannou F. (2017). The safety of intra-articular injections for the treatment of knee osteoarthritis: A critical narrative review. Expert Opin. Drug Saf..

[114] Gil C., Abdoul H., Campagna R., Guerini H., Ieong E., Chagny F., Bedin C., Roren A., Lefèvre-Colau M.M., Poiraudeau S. (2018). Intra-articular botulinum toxin A for base-of-thumb osteoarthritis: Protocol for a randomised trial (RHIBOT). BMJ Open.

[115] Van Daele D.J., Finnegan E.M., Rodnitzky R.L., Zhen W., McCulloch T.M., Hoffman H.T. (2002). Head and neck muscle spasm after radiotherapy: Management with botulinum toxin A injection. Arch. Otolaryngol. Head Neck Surg..

[116] Mailly M., Benzakin S., Chauvin A., Brasnu D., Ayache D. (2019). Radiation-induced head and neck pain: Management with botulinum toxin a injections. Cancer Radiother..

[117] Shaw L., Bazzell A.F., Dains J.E. (2019). Botulinum Toxin for Side-Effect Management and Prevention of Surgical Complications in Patients Treated for Head and Neck Cancers and Esophageal Cancer. J. Adv. Pract. Oncol..

[118] Lovato A., Restivo D.A., Ottaviano G., Marioni G., Marchese-Ragona R. (2017). Botulinum toxin therapy: Functional silencing of salivary disorders. Acta Otorhinolaryngol. Ital..

[119] Xie Y., Zhou J., Li H., Cheng C., Herrler T., Li Q. (2014). Classification of masseter hypertrophy for tailored botulinum toxin type A treatment. Plast. Reconstr. Surg..

[120] Melville J.C., Stackowicz D.J., Jundt J.S., Shum J.W. (2016). Use of Botox (OnabotulinumtoxinA) for the Treatment of Parotid Sialocele and Fistula After Extirpation of Buccal Squamous Cell Carcinoma With Immediate Reconstruction Using Microvascular Free Flap: A Report of 3 Cases. J. Oral. Maxillofac. Surg..

[121] Boukovalas S., Mays A.C., Selber J.C. (2019). Botulinum Toxin Injection for Lower Face and Oral Cavity Raynaud Phenomenon After Mandibulectomy, Free Fibula Reconstruction, and Radiation Therapy. Ann. Plast. Surg..

[122] Ulloa F., Gonzalez-Junca A., Meffre D., Barrecheguren P.J., Martinez-Marmol R., Pazos I., Olivé N., Cotrufo T., Seoane J., Soriano E. (2015). Blockade of the SNARE protein syntaxin 1 inhibits glioblastoma tumor growth. PLoS ONE.

[123] He D., Manzoni A., Florentin D., Fisher W., Ding Y., Lee M., Ayala G. (2016). Biologic effect of neurogenesis in pancreatic cancer. Hum. Pathol..

[124] Matak I., Lackovic Z. (2015). Botulinum neurotoxin type A: Actions beyond SNAP-25?. Toxicology.

[125] Bandala C., Perez-Santos J.L., Lara-Padilla E., Delgado Lopez G., Anaya-Ruiz M. (2013). Effect of botulinum toxin A on proliferation and apoptosis in the T47D breast cancer cell line. Asian Pac. J. Cancer Prev..

[126] Jain N., Lansiaux E., Yucel U., Huenermund S., Goeschl S., Schlagenhauf P. (2023). Outbreaks of iatrogenic botulism in Europe: Combating off-label medical use of Botulinum Neurotoxin (BoNT) in bariatric procedures. New Microbes New Infect..

[127] Lacy D.B., Tepp W., Cohen A.C., DasGupta B.R., Stevens R.C. (1998). Crystal structure of botulinum neurotoxin type A and implications for toxicity. Nat. Struct. Biol..

[128] Kumaran D., Eswaramoorthy S., Furey W., Navaza J., Sax M., Swaminathan S. (2009). Domain organization in Clostridium botulinum neurotoxin type E is unique: Its implication in faster translocation. J. Mol. Biol..

[129] Chaddock J.A., Marks P.M. (2006). Clostridial neurotoxins: Structure-function led design of new therapeutics. Cell. Mol. Life Sci..

[130] Fonfria E., Elliott M., Beard M., Chaddock J.A., Krupp J. (2018). Engineering Botulinum Toxins to Improve and Expand Targeting and SNARE Cleavage Activity. Toxins.

[131] Pirazzini M., Rossetto O., Eleopra R., Montecucco C. (2017). Botulinum Neurotoxins: Biology, Pharmacology, and Toxicology. Pharmacol. Rev..

[132] Elliott M., Favre-Guilmard C., Liu S.M., Maignel J., Masuyer G., Beard M., Boone C., Carré D., Kalinichev M., Lezmi S. (2019). Engineered botulinum neurotoxin B with improved binding to human receptors has enhanced efficacy in preclinical models. Sci. Adv..

[133] Foran P.G., Mohammed N., Lisk G.O., Nagwaney S., Lawrence G.W., Johnson E., Smith L., Aoki K.R., Dolly J.O. (2003). Evaluation of the therapeutic usefulness of botulinum neurotoxin B, C1, E, and F compared with the long lasting type A. Basis for distinct durations of inhibition of exocytosis in central neurons. J. Biol. Chem..

[134] Tsai Y.C., Kotiya A., Kiris E., Yang M., Bavari S., Tessarollo L., Oyler G.A., Weissman A.M. (2017). Deubiquitinating enzyme VCIP135 dictates the duration of botulinum neurotoxin type A intoxication. Proc. Natl. Acad. Sci. USA.

[135] Kohda T., Nakamura K., Hosomi K., Torii Y., Kozaki S., Mukamoto M. (2017). Response to “Standardized methods must be used to compare the properties of botulinum toxin serotypes”. Microbiol. Immunol..

[136] Eleopra R., Rinaldo S., Montecucco C., Rossetto O., Devigili G. (2020). Clinical duration of action of different botulinum toxin types in humans. Toxicon.

[137] Kutschenko A., Reinert M.C., Krez N., Liebetanz D., Rummel A. (2017). BoNT/AB hybrid maintains similar duration of paresis as BoNT/A wild-type in murine running wheel assay. Neurotoxicology.

[138] Malik S., Boissy R.E., Brideau-Andersen A., Sondergaard B. (2024). Sondergaard editor Botulinum Neurotoxin Type DC (BoNT/DC) Blocks Melanocyte Melanogenesis. Toxins.

[139] Whitemarsh R.C., Tepp W.H., Johnson E.A., Pellett S. (2014). Persistence of botulinum neurotoxin a subtypes 1–5 in primary rat spinal cord cells. PLoS ONE.

[140] Moritz M.S., Tepp W.H., Inzalaco H.N., Johnson E.A., Pellett S. (2019). Comparative functional analysis of mice after local injection with botulinum neurotoxin A1, A2, A6, and B1 by catwalk analysis. Toxicon.

[141] Pier C.L., Chen C., Tepp W.H., Lin G., Janda K.D., Barbieri J.T., Pellett S., Johnson E.A. (2011). Botulinum neurotoxin subtype A2 enters neuronal cells faster than subtype A1. FEBS Lett..

[142] Rasetti-Escargueil C., Avril A., Chahboun S., Tierney R., Bak N., Miethe S., Mazuet C., Popoff M.R., Thullier P., Hust M. (2015). Development of human-like scFv-Fc antibodies neutralizing Botulinum toxin serotype B. MAbs.

[143] Tepp W.H., Lin G., Johnson E.A. (2012). Purification and characterization of a novel subtype a3 botulinum neurotoxin. Appl. Environ. Microbiol..

[144] Davies J.R., Rees J., Liu S.M., Acharya K.R. (2018). High resolution crystal structures of Clostridium botulinum neurotoxin A3 and A4 binding domains. J. Struct. Biol..

[145] Tian R., Widel M., Imanian B. (2022). The Light Chain Domain and Especially the C-Terminus of Receptor-Binding Domain of the Botulinum Neurotoxin (BoNT) Are the Hotspots for Amino Acid Variability and Toxin Type Diversity. Genes.

[146] Henkel J.S., Jacobson M., Tepp W., Pier C., Johnson E.A., Barbieri J.T. (2009). Catalytic properties of botulinum neurotoxin subtypes A3 and A4. Biochemistry.

[147] Tepp W.H., Bradshaw M., Gardner A.P., Kaufman R.L., Barbieri J.T., Pellett S. (2023). Botulinum Neurotoxin A4 Has a 1000-Fold Reduced Potency Due to Three Single Amino Acid Alterations in the Protein Receptor Binding Domain. Int. J. Mol. Sci..

[148] Yin L., Masuyer G., Zhang S., Zhang J., Miyashita S.I., Burgin D., Lovelock L., Coker S.F., Fu T.M., Stenmark P. (2020). Characterization of a membrane binding loop leads to engineering botulinum neurotoxin B with improved therapeutic efficacy. PLoS Biol..

[149] Steward L., Brin M.F., Brideau-Andersen A. (2020). Novel Native and Engineered Botulinum Neurotoxins. Handbook of Experimental Pharmacology.

[150] Dolly J.O., Wang J., Zurawski T.H., Meng J. (2011). Novel therapeutics based on recombinant botulinum neurotoxins to normalize the release of transmitters and pain mediators. FEBS J..

[151] Wang J., Meng J., Lawrence G.W., Zurawski T.H., Sasse A., Bodeker M.O., Gilmore M.A., Fernández-Salas E., Francis J., Steward L.E. (2008). Novel chimeras of botulinum neurotoxins A and E unveil contributions from the binding, translocation, and protease domains to their functional characteristics. J. Biol. Chem..

[152] Dolly J.O., O’Connell M.A. (2012). Neurotherapeutics to inhibit exocytosis from sensory neurons for the control of chronic pain. Curr. Opin. Pharmacol..

[153] Wang J., Casals-Diaz L., Zurawski T., Meng J., Moriarty O., Nealon J., Edupuganti O.P., Dolly O. (2017). A novel therapeutic with two SNAP-25 inactivating proteases shows long-lasting anti-hyperalgesic activity in a rat model of neuropathic pain. Neuropharmacology.

[154] Elliott M., Maignel J., Liu S.M., Favre-Guilmard C., Mir I., Farrow P., Hornby F., Marlin S., Palan S., Beard M. (2017). Augmentation of VAMP-catalytic activity of botulinum neurotoxin serotype B does not result in increased potency in physiological systems. PLoS ONE.

[155] Wang D., Zhang Z., Dong M., Sun S., Chapman E.R., Jackson M.B. (2011). Syntaxin requirement for Ca^2+^-triggered exocytosis in neurons and endocrine cells demonstrated with an engineered neurotoxin. Biochemistry.

[156] Zanetti G., Sikorra S., Rummel A., Krez N., Duregotti E., Negro S., Henke T., Rossetto O., Binz T., Pirazzini M. (2017). Botulinum neurotoxin C mutants reveal different effects of syntaxin or SNAP-25 proteolysis on neuromuscular transmission. PLoS Pathog..

[157] Pirazzini M., Rossetto O., Bertasio C., Bordin F., Shone C.C., Binz T., Montecucco C. (2013). Time course and temperature dependence of the membrane translocation of tetanus and botulinum neurotoxins C and D in neurons. Biochem. Biophys. Res. Commun..

[158] Shone C.C., Hambleton P., Melling J. (1987). A 50-kDa fragment from the NH2-terminus of the heavy subunit of Clostridium botulinum type A neurotoxin forms channels in lipid vesicles. Eur. J. Biochem..

[159] Shone C.C., Hambleton P., Melling J. (1985). Inactivation of Clostridium botulinum type A neurotoxin by trypsin and purification of two tryptic fragments. Proteolytic action near the COOH-terminus of the heavy subunit destroys toxin-binding activity. Eur. J. Biochem..

[160] Chaddock J.A., Purkiss J.R., Friis L.M., Broadbridge J.D., Duggan M.J., Fooks S.J., Shone C.C., Quinn C.P., Foster K.A. (2000). Inhibition of vesicular secretion in both neuronal and nonneuronal cells by a retargeted endopeptidase derivative of Clostridium botulinum neurotoxin type A. Infect. Immun..

[161] Chaddock J.A., Herbert M.H., Ling R.J., Alexander F.C., Fooks S.J., Revell D.F., Quinn C.P., Shone C.C., Foster K.A. (2002). Expression and purification of catalytically active, non-toxic endopeptidase derivatives of Clostridium botulinum toxin type A. Protein Expr. Purif..

[162] Foster K.A., Adams E.J., Durose L., Cruttwell C.J., Marks E., Shone C.C., Chaddock J.A., Cox C.L., Heaton C., Sutton J.M. (2006). Re-engineering the target specificity of Clostridial neurotoxins—A route to novel therapeutics. Neurotox. Res..

[163] Darios F., Niranjan D., Ferrari E., Zhang F., Soloviev M., Rummel A., Bigalke H., Suckling J., Ushkaryov Y., Naumenko N. (2010). SNARE tagging allows stepwise assembly of a multimodular medicinal toxin. Proc. Natl. Acad. Sci. USA.

[164] Ferrari E., Maywood E.S., Restani L., Caleo M., Pirazzini M., Rossetto O., Hastings M.H., Niranjan D., Schiavo G., Davletov D. (2011). Re-assembled botulinum neurotoxin inhibits CNS functions without systemic toxicity. Toxins.

[165] Nugent M., Yusef Y.R., Meng J., Wang J., Dolly J.O. (2018). A SNAP-25 cleaving chimera of botulinum neurotoxin/A and /E prevents TNFalpha-induced elevation of the activities of native TRP channels on early postnatal rat dorsal root ganglion neurons. Neuropharmacology.

[166] Chen S., Barbieri J.T. (2009). Engineering botulinum neurotoxin to extend therapeutic intervention. Proc. Natl. Acad. Sci. USA.

[167] Pavlik B.J., Hruska E.J., Van Cott K.E., Blum P.H. (2016). Retargeting the Clostridium botulinum C2 toxin to the neuronal cytosol. Sci. Rep..

[168] Vazquez-Cintron E.J., Beske P.H., Tenezaca L., Tran B.Q., Oyler J.M., Glotfelty E.J., Angeles C.A., Syngkon A., Mukherjee J., Kalb S.R. (2017). Engineering Botulinum Neurotoxin C1 as a Molecular Vehicle for Intra-Neuronal Drug Delivery. Sci. Rep..

[169] Tang M., Meng J., Wang J. (2019). New Engineered-Botulinum Toxins Inhibit the Release of Pain-Related Mediators. Int. J. Mol. Sci..

[170] Miyashita S.I., Karatsu S., Fujiishi M., Huang I.H., Nagashima Y., Morobishi T., Hosoya K., Hata T., Dong M., Sagane Y. (2023). Characterization of Serotype CD Mosaic Botulinum Neurotoxin in Comparison with Serotype C and A. Toxins.

[171] Leese C., Bresnahan R., Doran C., Simsek D., Fellows A.D., Restani L., Caleo M., Schiavo G., Mavlyutov T., Henke T. (2020). Duplication of clostridial binding domains for enhanced macromolecular delivery into neurons. Toxicon X.

[172] Leese C., Christmas C., Meszaros J., Ward S., Maiaru M., Hunt S.P., Davletov B. (2023). New botulinum neurotoxin constructs for treatment of chronic pain. Life Sci. Alliance.

[173] Liu Z., Lee P.G., Krez N., Lam K.H., Liu H., Przykopanski A., Chen P., Yao G., Zhang Z., Tremblay J.M. (2023). Structural basis for botulinum neurotoxin E recognition of synaptic vesicle protein 2. Nat. Commun..

[174] Pons L., Vilain C., Volteau M., Picaut P. (2019). Safety and pharmacodynamics of a novel recombinant botulinum toxin E (rBoNT-E): Results of a phase 1 study in healthy male subjects compared with abobotulinumtoxinA (Dysport^®^). J. Neurol. Sci..

[175] Canty D., Nicholson G.S., Brideau-Andersen A.D., Broide R.S. (2024). Evaluation of Botulinum Neurotoxin type E preparation (TrenibotulinumtoxinE) in the mouse digit abduction score (DAS) assay. Toxicon.

[176] Machicoane M., Tonellato M., Zainotto M., Onillon P., Stazi M., Dal Corso M., Megighian A., Rossetto O., Le Doussal J.M., Pirazzini M. (2024). Excitation-Contraction Coupling Inhibitors Potentiate the Activity of Botulinum Neurotoxin Type A at the Neuromuscular Junction. Toxicon.

[177] Masuyer G., Stenmark P. (2024). Structure-Based Design of a Botulinum Neurotoxin With Triple Receptor Recognition. Toxicon.

[178] Krhut J., Navratilova M., Sykora R., Jurakova M., Gartner M., Mika D., Pavliska L., Zvara P. (2016). Intravesical instillation of onabotulinum toxin A embedded in inert hydrogel in the treatment of idiopathic overactive bladder: A double-blind randomized pilot study. Scand. J. Urol..

[179] Malek-Khatabi A., Rad-Malekshahi M., Shafiei M., Sharifi F., Motasadizadeh H., Ebrahiminejad V., Rad-Malekshahi M., Akbarijavar H., Rad Z.F. (2023). Botulinum toxin A dissolving microneedles for hyperhidrosis treatment: Design, formulation and in vivo evaluation. Biomater. Sci..

[180] Kaji R. (2023). A look at the future-new BoNTs and delivery systems in development: What it could mean in the clinic. Toxicon.

[181] Allen D., Hanumantharao S.N., McDonell R., Irvine K.A., Sahbaie P., Clark D., Blum P. (2023). Preclinical characterization of the efficacy and safety of biologic N-001 as a novel pain analgesic for post-operative acute pain treatment. Sci Rep..

[182] Liu K., Wang L. (2019). Optogenetics: Therapeutic spark in neuropathic pain. Bosn. J. Basic Med. Sci..

[183] Joussain C., Le Coz O., Pichugin A., Marconi P., Lim F., Sicurella M., Salonia A., Montorsi F., Wandosell F., Foster K. (2019). Botulinum Neurotoxin Light Chains Expressed by Defective Herpes Simplex Virus Type-1 Vectors Cleave SNARE Proteins and Inhibit CGRP Release in Rat Sensory Neurons. Toxins.

[184] Zeleny R., Busschots K., Kampa B., Worbs S., Skiba M., Dorner B., Van Nieuwenhuysen T., Puustinen A., Rasetti-Escargueil C., Nahori M. (2023). P20-01: First certified reference materials (CRMs) to improve the reliability of biological toxins detection, identification and quantification. Toxicol. Lett..

